# Differential response of placental cells to high D‐glucose and its impact on extracellular vesicle biogenesis and trafficking via small GTPase Ras‐related protein RAB‐7A

**DOI:** 10.1002/jex2.135

**Published:** 2024-01-15

**Authors:** Carlos Palma, Andrew Lai, Katherin Scholz‐Romero, Haarika Chittoory, Benjamin Van Haeringen, Flavio Carrion, Aase Handberg, Martha Lappas, Sunil R Lakhani, Amy E McCart Reed, H. David McIntyre, Soumyalekshmi Nair, Carlos Salomon

**Affiliations:** ^1^ Translational Extracellular Vesicles in Obstetrics and Gynae‐Oncology Group, Faculty of Medicine, University of Queensland Centre for Clinical Research, Royal Brisbane and Women's Hospital The University of Queensland Brisbane Queensland Australia; ^2^ UQ Centre for Clinical Research, Faculty of Medicine The University of Queensland Brisbane Australia; ^3^ Pathology Queensland The Royal Brisbane and Women's Hospital Brisbane Australia; ^4^ Departamento de Investigación, Postgrado y Educación Continua (DIPEC), Facultad de Ciencias de la Salud Universidad del Alba Santiago Chile; ^5^ Department of Clinical Biochemistry Aalborg University Hospital Aalborg Denmark; ^6^ Obstetrics, Nutrition and Endocrinology Group, Department of Obstetrics and Gynaecology University of Melbourne Victoria Australia; ^7^ Mercy Perinatal Research Centre Mercy Hospital for Women Victoria Australia; ^8^ Department of Obstetric Medicine, Mater Health Brisbane, Queensland and Mater Research The University of Queensland South Brisbane Queensland Australia

**Keywords:** biogenesis, extracellular vesicles, high glucose, MRM^HR^, trafficking

## Abstract

Placental extracellular vesicles (EVs) can be found in the maternal circulation throughout gestation, and their concentration, content and bioactivity are associated with pregnancy outcomes, including gestational diabetes mellitus (GDM). However, the effect of changes in the maternal microenvironment on the mechanisms associated with the secretion of EVs from placental cells remains to be fully established. Here, we evaluated the effect of high glucose on proteins associated with the trafficking and release of different populations of EVs from placental cells. BeWo and HTR8/SVneo cells were used as placental models and cultured under 5‐mM D‐glucose (i.e. control) or 25‐mM D‐glucose (high glucose). Cell‐conditioned media (CCM) and cell lysate were collected after 48 h. Different populations of EVs were isolated from CCM by ultracentrifugation (i.e. pellet 2K‐g, pellet 10K‐g, and pellet 100K‐g) and characterised by Nanoparticle Tracking Analysis. Quantitative proteomic analysis (IDA/SWATH) and multiple reaction monitoring protocols at high resolution (MRM^HR^) were developed to quantify 37 proteins related to biogenesis, trafficking/release and recognition/uptake of EVs. High glucose increased the secretion of total EVs across the pellets from BeWo cells, an effect driven mainly by changes in the small EVs concentration in the CCM. Interestingly, no effect of high glucose on HTR8/SVneo cells EVs secretion was observed. High glucose induces changes in proteins associated with vesicle trafficking in BeWo cells, including Heat Shock Protein Family A (Hsp70) Member 9 (HSPA9) and Member 8 (HSPA8). For HTR8/SVneo, altered proteins including prostaglandin F2α receptor regulatory protein (FPRP), RAB5A, RAB35, RAB5B, and RB11B, STAM1 and TSG101. These proteins are associated with the secretion and trafficking of EVs, which could explain in part, changes in the levels of circulating EVs in diabetic pregnancies. Further, we identified that proteins RAB11B, PDCD6IP, STAM, HSPA9, HSPA8, SDCBP, RAB5B, RAB5A, RAB7A and ERAP1 regulate EV release in response to high and low glucose when overexpressed in cells. Interestingly, immunohistochemistry analysis of RAB7A revealed distinct changes in placental tissues obtained from women with normal glucose tolerance (NGT, *n* = 6) and those with GDM (*n* = 6), influenced by diet or insulin treatment. High glucose regulation of proteins involved in intercellular dynamics and the trafficking of multivesicular bodies to the plasma membrane in placental cells is relevant in the context of GDM pregnancies.

## 1 INTRODUCTION

The establishment of maternal–foetal communication is one of the most crucial elements during pregnancy, and its main purpose is to ensure a successful pregnancy by orchestrating the correct implantation and early development of the placenta. However, communication can be profoundly disturbed by factors affecting placentation, which can lead to the development of pregnancy complications (Baschat, 2011; Brosens et al., 2011; Burton et al., 2009; Jauniaux et al., 2003; Jauniaux et al., 2006). Multiple factors, such as the immune system, enzymes, cytokines and messenger molecules, need to interact in a delicate balance. In this context, special attention has been sparked by the extracellular *milieu* and its effect on cellular adaptation. A further understanding of the potential influence of the cellular microenvironment is vital to contextualising the development of pregnancy complications and their repercussions. For instance, the postpartum development of diabetes has been linked to pre‐exposure to a high level of glucose during pregnancy and previous metabolic status (Cosson et al., 2017), a phenomenon that can considerably affect the Australia population, where around 15% of pregnancies are associated with gestational diabetes mellitus (GDM) (Moses et al., 2011).

Our understanding of the active mechanisms in placental cells involved in pregnancy adaptations and their response to high glucose concentrations remains limited. Studies have shown that trophoblast cells exposed to high glucose exhibit the downregulation of cGMP‐dependent PKG, which may contribute to the vascular complications observed in diabetic mothers (Nguyen et al., 2015). Pathological changes in placental tissues have also been linked to issues with trophoblast viability and proliferation, indicating the impact of high glucose on the miR‑137/PRKAA1/IL‑6 axis (Peng et al., 2018). Furthermore, research has highlighted alterations in genes related to the cell cycle and metabolism under high glucose conditions (Inadera et al., 2010). Additionally, high glucose affects PKCβ and HIF‐1α pathways, potentially leading to the dysregulation of other angiogenic factors and the association of GDM with complications like preeclampsia (Mitsui et al., 2018).

The maternal–foetal communication involves signalling molecules, such as cytokines, during the differentiation and developmental processes (Zhao et al., 2019), modulation of the immune system (Pope, 1990; Rambaldi et al., 2019) and vascular remodelling (Burton & Jauniaux, 2018; Burton et al., 2009). However, in addition to cell‐derived signalling molecules or direct cell‐to‐cell communication, extracellular vesicles (EVs) have been recognised as important mediators of cell‐to‐cell communication. They are involved in delivering whole packages of bioactive molecules and signals to distant cells (O'Brien et al., 2020; Salomon et al., 2022; Veziroglu & Mias, 2020). Molecules within EVs are protected from the external environment and possible degradation (Batagov & Kurochkin, 2013; Cheng et al., 2014; Klibi et al., 2009), and EVs are secreted in most body fluids (e.g. blood, urine, saliva, tears and amniotic fluid) by various cell types, including but not limited to stem cells, immune cells, neurons and importantly, cells related to the development of a pathological condition (Kalluri, 2016; Zheng et al., 2018). Another interesting discovery was that the release of these vesicles can be modulated by other extracellular signals or molecules (e.g. histamine receptor agonist, Ca^2+^, respiratory syncytial virus), thus modifying the release rate or the cargo (Chahar et al., 2018; Savina et al., 2005; Verweij et al., 2018). However, the effect of a diabetic environment (e.g. high glucose) on the release and function of EVs is still unknown.

EVs refer to a diverse group of lipid bilayer‐delimited particles released by living cells into biological fluids. To categorize EVs, the International Society of Extracellular Vesicles (ISEV) recommends using size as a criterion (Thery et al., 2018). This classification includes small EVs (sEVs) (Colombo et al., 2014), encompassing exosomes and ectosomes, and large EVs, which consist of ectosomes, microvesicles and apoptotic bodies (Caruso & Poon, 2018; Chargaff & West, 1946; Colombo et al., 2014; Poutsiaka et al., 1985).

Different proteins are involved in biogenesis, trafficking/release and uptake/recognition of these vesicles. Classical biogenesis mechanisms identify Endosomal Sorting Complex Required for Transport (ESCRT) machinery as a crucial and one of the best‐described mechanisms which coordinate around 20 proteins grouped into four complexes and a few accessory proteins (Colombo et al., 2013; Henne et al., 2011). Hepatocyte growth factor‐regulated tyrosine kinase substrate (HRS) and signal transducing adaptor molecules (STAM) are members of complex ESCRT‐0 responsible for recruiting ubiquitinated cargo (Piper & Luzio, 2007). TSG101, VPS23 and VPS28 are members of ESCRT‐I, and they interact with ESCRT‐0 but also with components of ESCRT‐II such as VSP22, and VPS36, in order to promote membrane deformation and consequently, bud formation. ESCRT‐III, which comprehends proteins such as Charged multivesicular body protein 4c (CHMP4c) or CHMP6, facilitates the scission of the bud and the accessory proteins (Alix, Bro1) are necessary to dissociate the complexes and recycle protein components (Hurley, 2010). In terms of trafficking of EVs, several members of the Rab family have been identified as key players in the mobilisation of vesicles in the cell, allowing their release to the extracellular space (Baietti et al., 2012), whereas N‐ethylmaleimide‐sensitive factor attachment protein receptors or SNARE, such as VAMP7, have been involved in the fusion of vesicles and different targeted membranes (Bonifacino & Glick, 2004). The interaction between EV‐target cells is mediated by a variety of receptors, such as tetraspanins. These proteins are a highly conserved family of membrane palmitoylated proteins that regulate several processes in the cell such as motility, plasma membrane interactions and protein trafficking (Boismenu et al., 1996; Boucheix & Rubinstein, 2001; Hemler, 2008). CD9, CD81 and CD63 are some of the tetraspanins enriched in EVs, reason why they are used as markers to define which sub‐population of EVs are being isolated and they are part of the proteins used in characterisation according to the Guidelines of the International Society of Extracellular Vesicles 2018 (Escola et al., 1998; Thery et al., 2018).

The process of biogenesis leads to the formation of approximately 30–150‐nm particles, and evidence also suggests a regulated mechanism of packaging for several proteins and other nucleic acid molecules (Kowal et al., 2014), although the details of the mechanism remain unclear. These cargo molecules may be appointed by the cell of origin (Kobayashi et al., 2014) as a response that requires specific bioactivity to tissue physiological conditions. Early evidence supports the idea that sEVs are secreted in response to extracellular microenvironmental conditions such as oxygen tension and glucose concentration (Rice et al., 2015; Salomon et al., 2013; Salomon et al., 2013). In vitro experiments using first‐trimester trophoblast cells showed that the release rate, as well as the cargo and bioactivity of sEVs, can be modified by exposure of cells to different oxygen tensions (Salomon et al., 2013). In addition, it has been reported that sEVs isolated from a trophoblast cell line can promote processes such as cell migration and apoptosis in a different cell type (Salomon et al., 2014).

Furthermore, previous data have established the presence of EVs derived from trophoblast cells in maternal circulation as early as the 6th week of pregnancy (Sarker et al., 2014). Nevertheless, our understanding of the regulation exercised by the extracellular *milieu* on the release of this sub‐population of EVs remains limited. In order to have a better understanding of the effect of glucose on pregnancy, several studies have evaluated proteins and lipids related to the main types of machinery used by cells (Colombo et al., 2013; Devaux & Morris, 2004; Escola et al., 1998; Gross et al., 2012; Hoshino et al., 2013; Kinslechner et al., 2019; Tamai et al., 2010; Thery et al., 2018). Building on this concept, this study aimed to evaluate the effect of high glucose on the regulation of the release and proteomic content of various EV populations from in vitro placental models, specifically human villous (BeWo) and extravillous (HTR‐8/SVneo) trophoblast cells. The results of this study demonstrated a differential response of HTR‐8/SVneo and BeWo cells to glucose, which in turn regulates the proteins associated with the trafficking and secretion of distinct EV types. These findings could have implications for how placental cells sense the extracellular milieu during pregnancy to secrete EV that will influence the foetal maternal communication during gestation. However, it is important to highlight that while BeWo and HTR‐8 cell lines are valuable tools, they do not fully replicate the complexity of primary trophoblast cells in vivo. Therefore, data derived from these cell lines should be analysed with caution when interpreting results in the context of physiological conditions.

## 2 MATERIALS AND METHODS

### 2.1 Regulatory environment and data quality assurance

All experimental procedures were conducted within an ISO17025 accredited (National Association of Testing Authorities, Australia) research facility. All data were recorded within a 21 Code of Federal Regulation (CFR) part 11 compliant electronic laboratory notebook (Lab Archives, Carlsbad, CA 92008, USA).

### 2.2 Cell culture conditions

In this study, we used BeWo and extravillous HTR‐8/SVneo cell lines as placenta models, as we could not have access to primary placenta cells for the timeframe of this study. BeWo trophoblast cell line was purchased from the European Collection of Cell Cultures (Porton Down, Salisbury, United Kingdom), and was used as a placental cell model to analyse the effect in vitro of mimetic exposure to a hyperglycaemic microenvironment. These cells were cultured in Nutrient mixture F‐12 Ham (N3520, Sigma‐Aldrich) supplemented with 10% foetal bovine serum (FBS, 10437‐02, Gibco^®^) and 1% antimicotic/antibiotic (AA, 15240‐062, Gibco^®^), and maintained at 37°C in a humidified chamber, under an atmosphere of 5% CO_2_‐balanced N_2_ to obtain 8% O_2_ (pO_2_ ∼54 mmHg) in a multi‐gas incubator (Panasonic MCO‐19 M‐PE). In addition, HTR8/SVneo were used for the assessment of D‐glucose. HTR8/SVneo cells were cultured in the Roswell Park Memorial Institute 1640 (RPMI) medium (11835‐030, Gibco^®^), supplemented with 10% FBS and 1% AA. Cells were sub‐cultured with dissociation media, TrypLE™ Express (Life Technologies). For both cell lines, D‐glucose treatment was performed in triplicate and over 48 h, in an FBS‐free culture medium. For D‐glucose treatment (K023‐021, Gibco^®^), growth media was considered the normal condition whereas, for high glucose, 20‐mM glucose was added. HEK293T cells were used for plasmid transfection and analyse the effect of protein on EV biogenesis. HEK293T cells were grown in Dulbecco's modified Eagle's medium (DMEM), supplemented with 10% FBS and 1% AA. To confirm the authenticity and origin of BeWo and HTR8/SVneo cell lines, Short Tandem Repeat (STR) profiling was conducted, utilising a panel of highly polymorphic genetic markers, following established protocols for cell line authentication and CLASTR: The Cellosaurus STR similarity search tool (Robin et al., 2020) (Figure S1), which confirm the authenticity of Bewo and HTR8/SVneo cells.

### 2.3 Isolation of EVs

EVs were isolated from 60 mL of cell‐conditioned media (CCM) (equivalent to around 50 × 10^6^ cells) by differential centrifugation, followed by ultracentrifugation. In brief, CCM was centrifuged at 2000 × *g* for 20 min in 50‐mL conical tubes (352070, Falcon) (pellet 1 = 2K pellet). The supernatant was transferred to a new tube, and the pellet was re‐suspended in 100 μL of filtered PBS (14190‐144, Gibco^®^). The supernatant was centrifuged at 10,000 × *g* for 40 min. The supernatant was then transferred to ultracentrifugation polycarbonate bottles (355651, Beckman Coulter) and the pellet was re‐suspended in 100 μL of filtered PBS (pellet 2 = 10K pellet). The supernatant was centrifuged at 100,000 × *g* for 90 min in a Type 70.1 Ti Fixed Angle Rotor (Thermo Fisher Scientific, MA, USA). The final pellet was re‐suspended in 100 μL of PBS (pellet 3 = 100K pellet). The pellets (1, 2 and 3) were stored at −80°C until further analysis.

### 2.4 Nanoparticle tracking analysis (NTA)

The size distribution and concentration of particles in each pellet was measured via NTA software (NTA 3.1 Build 3.1.46), in triplicate, using the NanoSight NS500 instrument (Malvern, UK), configured with a 405‐nm laser and a digital camera system (sCMOS). Samples were diluted with sterile‐filtered PBS to ensure a range of concentrations to obtain >200 tracks. After loading the first sample into the visualising area, the camera level 13 was set to obtain an image with sufficient contrast to identify the nanoparticles whilst minimizing background noise. The NTA software captured triplicate 30‐s video recordings of the EVs with the temperature of the laser unit controlled to 25°C. The videos were analysed by NTA 3.1, which translates the Brownian motion and light scatter properties of each individual laser‐illuminated particle into a size distribution (ranging from 5 to 2000 nm) and concentration (particles per millilitre).

### 2.5 Western Blot

For EV characterisation, 10 μg per sample was separated by polyacrylamide gel electrophoresis, transferred to Immobilon‐^®^FL polyvinylidene difluoride membrane (Millipore, Billerica, MA, USA) and blocked 1 h with buffer Odyssey^®^ Blocking Buffer (TBS) (927‐50000). Membranes were then probed with primary rabbit monoclonal anti‐CD9 (expected MW: 25 kDa; 1:1000; ab92726, Abcam), primary rabbit monoclonal anti‐TSG101 (expected MW: 44 kDa; 1:1000, ab125011, Abcam), primary rabbit monoclonal anti‐PLAP (expected MW: 58 kDa; 1:1000; ab133602, Abcam), rabbit monoclonal anti‐Grp94 (expected MW: 100 kDa; 1:1000; 20292T, Cell Signaling), anti‐FLAG (1:500; A00187, GenScript) and anti‐GAPDH (expected MW: 37 kDa; 1:1000; 5174, Cell Signaling). After at least 16 ho of incubation, membranes were washed three times for 10 min each with Tris buffer saline (TBS, 28358) – 0.1% Tween‐20 (P1379) (TBST). The membranes were then incubated for 1 hour in a blocking buffer containing antibody goat anti‐rabbit IgG (LCR‐925‐32212 IRDye 800CW, LI‐COR Biosciences). Next, membranes were washed three times, 10 min each, with TBST. The blots were analysed using the ChemiDoc MP Imaging System.

### 2.6 Mass spectrometry

#### 2.6.1 Protein quantification from lysate and extracellular vesicle samples

Proteins extracted from cells were quantified using the optimised protocol of the DC™ Protein Assay Kit II (5000112, Bio‐Rad). Colourimetric measures were performed in Tecan's Spark 20 M multimode microplate reader. Since EVs have a lower concentration of proteins, quantification was determined using the optimised protocol of the MicroBCA™ Protein Assay Kit (23235, Thermo Scientific).

### 2.7 Sample preparation

After determining the protein concentration, 40 μg of proteins were processed per sample. Filter‐aided sample preparation (FASP) was used for sample preparation. Briefly, lysates and EVs samples were mixed with lysis buffer (5% sodium dodecyl sulphate (SDS)/100‐mM dithiothreitol (DTT)/500‐mM ammonium bicarbonate (ABC)), sonicated and incubated for 1 h at room temperature. After the incubation, 50‐mM iodoacetic acid (IAA) was added and samples were incubated in the dark for 30 min. Samples were then diluted five times with a mix containing 8‐M urea/100‐mM ABC. Next, samples were transferred into filter tubes (Pall Nanosep^®^ Centrifugal Device with Omega membrane, MW cut‐off: 30 kDa, Sigma‐Aldrich), centrifuged and washed with the urea solution. An extra wash step with 50‐mM ABC was performed before incubating with 50‐μL diluted trypsin in ABC (enzyme to protein ratio, 1:50) at 37°C overnight. After overnight incubation, the filter membrane was transferred to low protein binding tubes. Peptides were centrifuged and followed by an extra 50‐μL ABC buffer wash, and then acidified using 10% trifluoroacetic acid (TFA) and desalted using the solid‐phase extraction SOLAμ™ SPE plate (THC60209‐001, ThermoFisher). The eluted tryptic products were completely dried in a vacuum concentrator and re‐suspended in 0.1% (v/v) formic acid (FA) before being transferred into screw thread glass autosampler vials (Thermo Fisher).

### 2.8 IDA/SWATH and MRM^HR^ protocols

A reversed‐phase trap column (CHROMXP C18‐CL 5 μm, 10 × 0.3 mm; Eksigent, Redwood City) was used to load the tryptic products resulting from the sample preparation, and separation was performed using a reversed‐phase analytical column (CHROMXP C18‐CL 3 μm, 15 cm × 0.075 mm; Eksigent, Redwood City). The loading phase was performed at a flow rate of 3 μL/min for 15 min of 0.1% FA/H_2_O (LC‐MS solvent A). After this step, the separation of the peptides was performed at a flow rate of 0.25 μL/min as follows. Time 0 started with 4.8% of 100% acetonitrile/0.1% FA (LC‐MS solvent B) and the gradient was increased to 8.8% Solvent B for 10 min. Then, a linear gradient was applied from 8.8 to 26% solvent B for 1 h, followed by a gradient from 26 to 40% solvent B for 15 min. The concentration of solvent B was gradually increased from 40 to 95.2% for 7 min and maintained at 95.2% solvent B for 12 min. Later, the concentration of solvent B in the column was decreased from 95.2 to 0.8% over 6 min. A final equilibration step was performed by running 0.8% solvent B for 10 min. The information dependent acquisition (IDA) was performed using an AB Sciex 5600 TripleTOF mass spectrometer where the top 40 precursor ions were automatically selected for fragmentation (TOF‐MS: 200‐ms accumulation time; product‐ion: 50‐ms accumulation time). In the case of SWATH acquisition, the same gradient conditions were used as outlined above and the isolation width of 26 Da (optimal ion transmission efficiency of 25 Da and window overlap of 1 Da) (Gillet et al., 2012) was applied to acquire the data.

The local ion library was generated by searching in SwissProt (Homo sapiens) database using Protein Pilot version 5.0 Software (ABSCIEX) and the Paragon Algorithm. The resulting SWATH data were analysed by using the SWATH Acquisition Microapp 2.0 in PeakView 2.2 (SCIEX) where the 1% global false discovery rate (FDR) was used as the threshold for the number of proteins for import. The processing settings for the SWATH analysis targeted four peptides per protein, three transitions per peptide, with a peptide confidence threshold of 99%, and FDR threshold of 1%. The retention time calibration was performed using a set of spiked‐in peptides (iRT) (Escher et al., 2012) distributed along the time axis. The peak area data were exported into MarkerView 1.3.1 (SCIEX) for statistical analysis and normalised by total area sum (TAS). Differences in protein expression between control and high glucose groups were compared using *t*‐tests (*p* < 0.05). For MRM^HR^, 37 proteins involved in the EV machinery (biogenesis, trafficking/release and uptake) were evaluated by developing a method on Skyline Software (MacCoss Lab, University of Washington, WA). This method considered at least three peptides per protein (exception for highly related protein where unique peptides were evaluated, see Table 1). Specific precursor was selected in each peptide. Information collected at transition level was analysed using MultiQuant 3.0 Software (SCIEX). The peak areas for each peptide are the result of the sum of the peak areas of the transition ions observed per peptide normalised by TAS. The data were analysed using GraphPad Prism version 9.0.0. (La Jolla, California, USA) and Qlucore Omics Explorer (Lund, Sweden).

**TABLE 1:** Size distribution (mean and mode) of the extracellular vesicles obtained for each pellet analysed from Bewo and HTR‐8 cells.

Mean nm						
	Bewo cells			HTR‐8/SVneo cells		
Pellets	5 mM	25 mM	*p*‐value	5 mM	25 mM	*p*‐value
2000 g	175.0 ± 39.3 (128.8–231.0)	133.8 ± 6.8 (126.3–148.0)	0.0069	237.9 ± 19.0 (219.4–282.9)	249.5 ± 16.1 (225.6–268.1)	0.1814
10,000 g	156.3 ± 16.0 (133.6–178.0)	108.5 ± 2.6 (104.4–112.0)	<0.0001	245.9 ± 18.7 (210.6–269.1)	242.0 ± 19.3 (214.7–269.7)	0.6691
100,000 g	168.2 ± 17.5 (149.7–198.0)	103.3 ± 1.2 (100.9–104.8)	<0.0001	170.1 ± 10.6 (158.0–184.7)	170.5 ± 13.5 (145.0–183.2)	0.9451
2000 g	112.7–14.4 (95.8 – 135.6)	107.1 ± 5.4 (97.8–118.2)	0.2908	168.3 ± 88.5 (102.2–311.3)	129.2 ± 38.6 (48.5–162.6)	0.2420
10,000 g	109.8 ± 8.3 (102.5–123.6)	94.5 ± 5.2 (86.0–102.3)	0.0002	145.7 ± 17.2 (119.8–166.5)	134.6 ± 27.0 (87.3–177.1)	0.3137
100,000 g	104.8 ± 5.4 (95.7–114.3)	92.0 ± 2.1 (88.3–94.3)	<0.0001	135.2 ± 20.1 (97.3–161.8)	128.3 ± 12.2 (106.5–150.5)	0.3917

### 2.9 Bioinformatics analysis

Differentially expressed proteins were analysed further by bioinformatic pathway analysis (ingenuity pathway analysis [IPA]; Ingenuity Systems, Mountain View, CA; www.ingenuity.com).

### 2.10 Plasmid preparation

Plasmids with inserts (HSPA8, ERAP1, HSPA9, RAB7A, PDCD6IP, SDCBP, RAB5A, STAM, RAB11B and RAB5B) cloned to vector pcDNA3.1+/C‐(K)‐DYK were purchased (GenEZORF clone, GeneScript). The plasmid DNA was transformed into One Shot Stbl3 chemically competent *E. coli* cells (Invitrogen) using heat shock method. Briefly, to 100 μL of competent cells 10 μg of plasmid was added and mixed gently. The mixture was incubated in ice for 30 min and then at 42°C for 45 s and incubated in ice for 2 min. Further, 1 mL of S.O.C medium was added to the transformed cells and incubated at 37°C with shaking at 150 rpm for 1 h. Spread 150 μL of transformation mixture on a LB agar with ampicillin plate and incubated at 37°C overnight. The colonies were selected and inoculated in 15 mL of LB broth with 100‐mg/mL ampicillin and incubated overnight. Plasmid isolation was performed using EZNA endo‐free plasmid DNA mini kit (Omega Bio‐Tek) using manufacturer's protocol. Briefly, bacterial cells were lysed using alkaline SDS lysis method. The cleared cell lysate is then treated with ETR reagent to remove the endotoxins. Subsequently, the cell lysate was applied into a HiBind DNA column and purified DNA was eluted from the column membrane. The DNA concentration in the plasmid preparation was measured using Nanodrop (Thermo Fisher Scientific).

### 2.11 Plasmid transfection

Plasmid transfection experiment was carried out in HEK293T cells. Approximately 2.3 × 10^5^ cells were seeded per well in 24 well plates and incubated overnight to achieve 60% confluency. Transfection was performed using 500 ngs plasmid/well, 1.5 μL of Lipofectamine 3000/well and P3000 reagent (Thermofisher) as per manufacturer's instruction. Prepared 5‐ and 25‐mM D‐glucose media in DMEM media without glucose (Gibco) and serum. After 48 h of transfection, the cells were washed and freshly prepared 5‐ and 25‐mM D‐glucose media was added. The transfected cells were incubated in the presence of 5‐ and 25‐mM glucose for 24 h and subsequently CCM were collected from the corresponding wells.

### 2.12 Immunohistochemistry (IHC)

The Research Ethics Committee of Mercy Health and The University of Queensland (HREC/09/QRBW/14) approved this study. Written, informed consent was obtained from all participating women. Human placentae were obtained from two groups of women who delivered singleton infants at term (≥37 weeks of gestation): (i) Group 1: Normal Glucose Tolerance test (NGT) where women, (ii) Group 2: GDM treated with diet and (iii) GDM treated with insulin. GDM was diagnosed by testing with a three sample 75 g OGTT at 24–28 weeks, with cut‐offs set according to ADIPS and WHO recommendations (Agarwal et al., 2014). Tissue samples were fixed in buffered formaldehyde solution (4%) and paraffin embedded for morphological and immunohistochemical analysis. IHC was performed on formalin‐fixed paraffin embedded 4‐μm placental sections using the MACH1 Universal HRP‐Polymer Detection Kit (BioCare Medical, Pacheco, CA, USA) according to the manufacturer's instructions. Briefly, the sections were de‐waxed and re‐hydrated using decreasing concentrations of alcohol (100%–70%). Heat‐ induced epitope retrieval was performed using sodium citrate buffer (0.01 M, pH 6.0) at 95°C for 30 min in a decloaking chamber (BioCare Medical) followed by treatment with 3% hydrogen peroxide for 10 min to remove endogenous peroxidases. The sections were blocked using the MACH1 sniper blocking reagent (BioCare Medical) and incubated with the primary antibodies: anti‐Rab7a (Sigma; HPA006964; 1:200; O/N at 4°C), anti‐Vimentin (Dako; M0725; 1:200, 1‐h RT), anti‐CK‐7 (Dako, M7018; 1:100, 1‐h RT) diluted in the DaVinci Green diluent (BioCare Medical) in a humidified slide chamber. MACH1 secondary antibody conjugated to horseradish peroxidase was applied to the sections for 30 min at room temperature, followed by diaminobenzidine (DAB) chromogenic substrate for 5 min. The slides were counterstained with Hematoxylin for 4 min and cover‐slipped using DPX mounting medium (Sigma Aldrich, St Louis, MO, USA). Sections were scored by a pathologist (BVH) and a Histo (H)‐score calculated (% of positive cells × staining intensity [low = 1; moderate = 2; high = 3]). We used *H*‐score as it is a widely used semi‐quantitative method in immunohistochemistry that help to assess the expression level of specific proteins in tissue samples, and it has been uniformly accepted as a valid pathology scoring tool since the mid‐1980s (Abd El‐Rehim et al., 2004; Goulding et al., 1995; McCarty et al., 1986; Rakha et al., 2014). Some of the advantages of using *H*‐score include reducing the subjectivity of valued scores, increase repeatability of scoring system, interpretation Consistency, and it has broader dynamic range (Fedchenko & Reifenrath, 2014; McCarty et al., 1985). The scoring pathologist in this study was blinded and serial sections were used for staining of orientating biomarker (CK7).

### 2.13 Statistical analysis

The effects of high glucose on the release of exosomes from placental cells are presented as the number of EVs released from 10^6^ cells/48 h (mean ± SE, *n* = 3–6 independent isolations). Differences were evaluated using the *t*‐test or ANOVA for parametric analysis, or Mann–Whitney *U* test for non‐parametric analysis. Statistical differences between groups were identified by *post hoc* analyses using Bonferroni's tests to compare each treatment to the control group where the data distribution approximates normality.

## 3 RESULTS

### 3.1 Characterisation of extracellular vesicles

**FIGURE 1:**
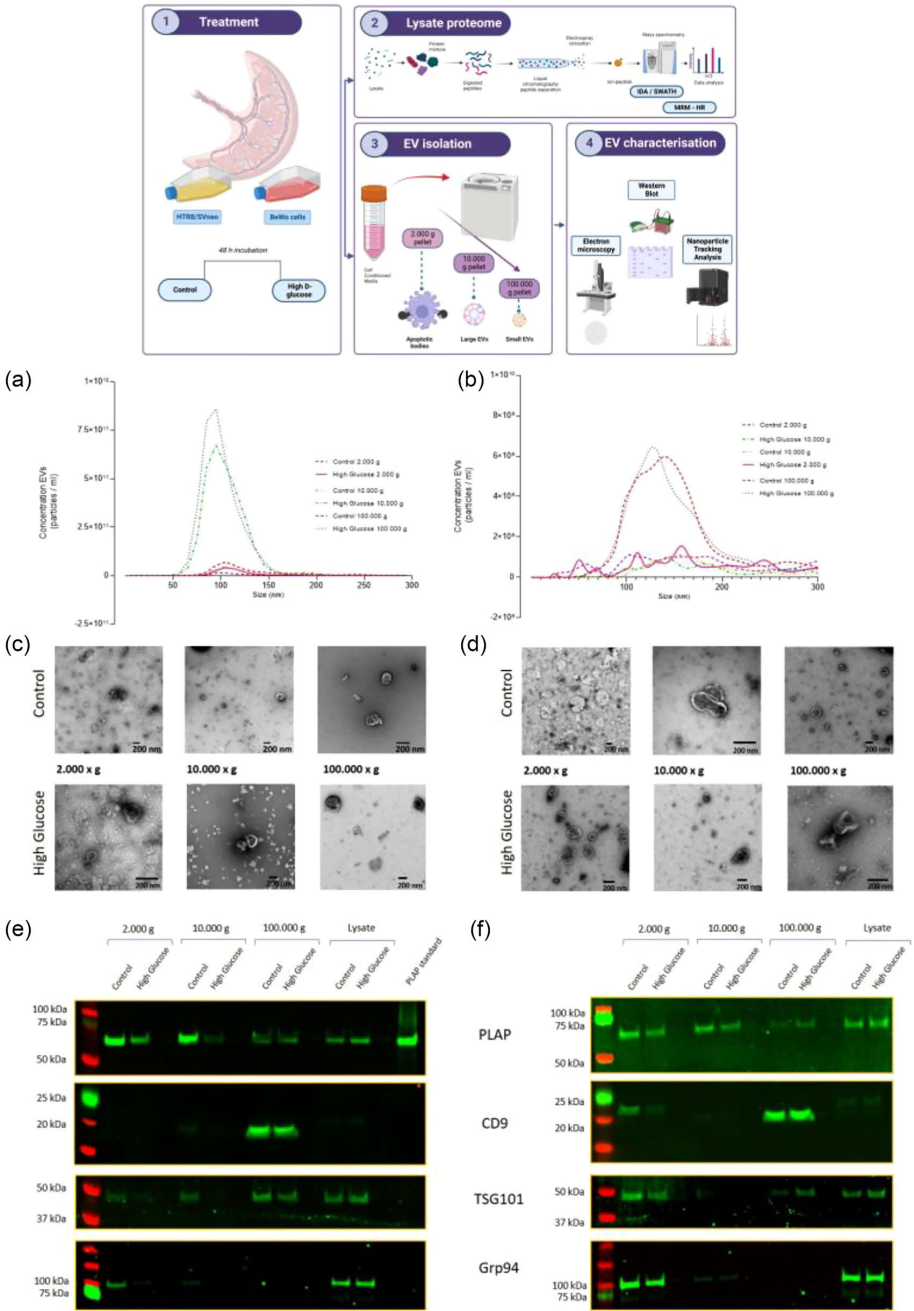
Characterisation of extracellular vesicles isolated from BeWo cells and HTR8/SVneo. Top: experimental design. NTA was performed in the three different pellets (2000, 10,000 and 100,000 g) isolated from (a) BeWo cells, and (b) HTR8/SVneo, control and high D‐glucose treatment. As part of the characterisation, TEM images were obtained for the same samples, (c) BeWo cells and (d) HTR8/Svneo. Western blot was performed to evaluate the expression of extracellular vesicle markers CD9 and TSG101, specific placental marker PLAP and cell control marker Grp94 using samples from (e) BeWo cells and (f) HTR8/SVneo.

Figure 1 (top) represents a flow diagram of the isolation method used to characterise different populations of EVs. BeWo cells, a cell line with properties related to villous trophoblast and HTR8/SVneo, a first‐trimester cell line with extravillous trophoblast features, were used in this study. We defined the different populations of EVs according to their size as small EVs (50–200 nm) and large EVs (>200 nm). Three different pellets were isolated based on centrifugation speed, that is, 2K × g, 10K × g and 100K × g. The isolated EVs were characterised by NTA, western blot (i.e. CD9 – ab92726, TSG101 – ab125011 and PLAP – ab133602) and transmission electron microscopy (TEM). The distribution of particles observed in the different curves for EVs isolated from BeWo cells, although differing in amplitude, presented a peak graphically situated around 100 nm (Figure 1a) whereas HTR8/SVneo EVs were observed to be around 130 nm for the 100K × g vesicles, but poorly defined in the other pellets (Figure 1b). Visual confirmation using TEM demonstrated the presence of EVs in representative samples obtained in the three different pellets for each cell line and condition tested. The classical small cup‐shaped morphology and lipid bilayer of EVs was observed (Figure 1c,d). The evaluation of markers of EVs by western blot revealed the presence of a strong signal for CD9 in the100K pellet in both EV samples, control and high glucose. A faint signal was observed in the 10K control and high glucose lysate samples. TSG101 was observed in the 100K sample and lysate, in both control and high glucose. Control 2K and control 10K showed a weaker signal. The placental specific marker, PLAP (placental alkaline phosphatase), was observed in all the pellets and lysates, regardless of whether they were control or high glucose samples (Figure 1e). A similar pattern was observed in vesicles isolated from HTR8/SVneo cells (Figure 1f), however, PLAP‐signal was slightly attenuated in all the samples, and Grp94 was shown to be highly present in the 2K pellet. Next, we evaluated the effect of high glucose on the secretion of different populations of EVs from BeWo and HTR8/SVneo cell lines.

### 3.2 Effect of high D‐glucose on the secretion of EV from placental cells

**FIGURE 2:**
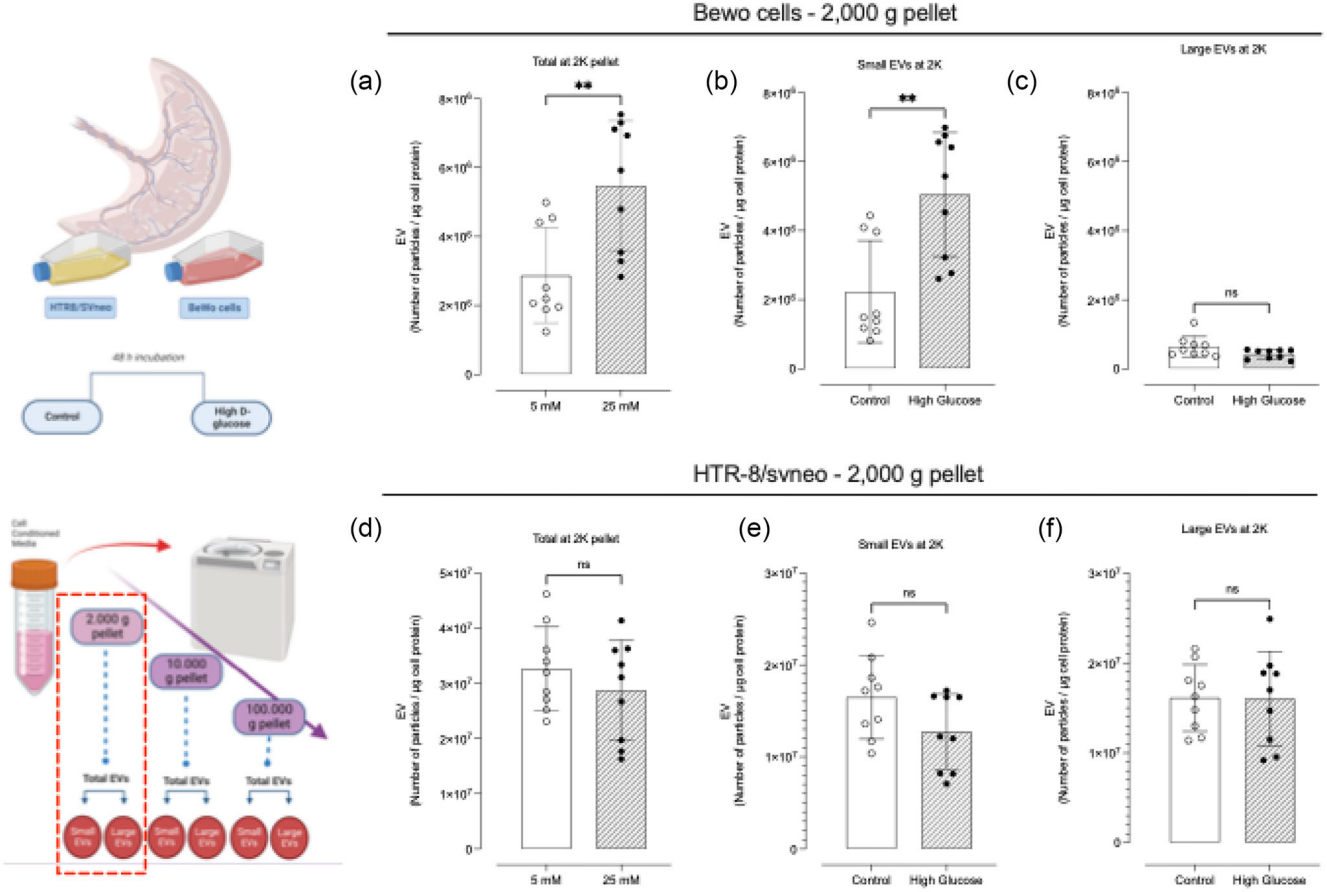
NTA of vesicles isolated at 2000 g from BeWo cells and HTR8/SVneo. Total number of particles observed in samples isolated at 2000 g after 20 min‐centrifugation, and the respective analysis by size distribution categorised as small and large EVs. Samples considered were (a)–(c) *BeWo cell* and (d)–(f) *HTR8/SVneo*.

To evaluate the effect of high glucose on the secretion of different populations of EV from placental cells, NTA was used to determine the size and concentration of the EVs in each pellet. High glucose (i.e. 25 mM) increased the secretion of total EVs from BeWo cells in each pellet. On the other hand, high glucose did not change the secretion of EVs from HTR8/SVneo cells. The concentration of total particles in the 2K pellet increased ∼1.9‐fold (*p*‐value = 0.0043) compared to control (i.e. 5 mM) (Figure 2a). Interestingly, the mean size of these vesicles decreased by 23.5% (*p* = 0.0003), from 175.0 to 133.8 nm, without changes in the mode (Table 1). Further, a sub‐classification of EVs by size was performed for each pellet. Analysis of the different populations categorised by size showed that the global changes observed in the 2K pellet was a result of the variation in the number of small EVs (<200 nm) released by BeWo cells in response to D‐glucose (Figure 2b,c). In the control condition, there were 1.9 × 10^6^ particles per μg of cell protein, whereas the concentration after treatment increased to 4.6 × 10^6^ particles per μg of cell protein (*p* = 0.0039). Analysis of the 2K pellet obtained from HTR8/SVneo showed no significant differences in the global number of particles released, nor in the specific subpopulations (Figure 2d–f). In terms of mean and mode, although the EVs from the HTR8/SVneo were bigger than EVs obtained from BeWo cells, there were no significant differences between control HTR8/SVneo and the same cells exposed to high glucose (Table 1).

**FIGURE 3:**
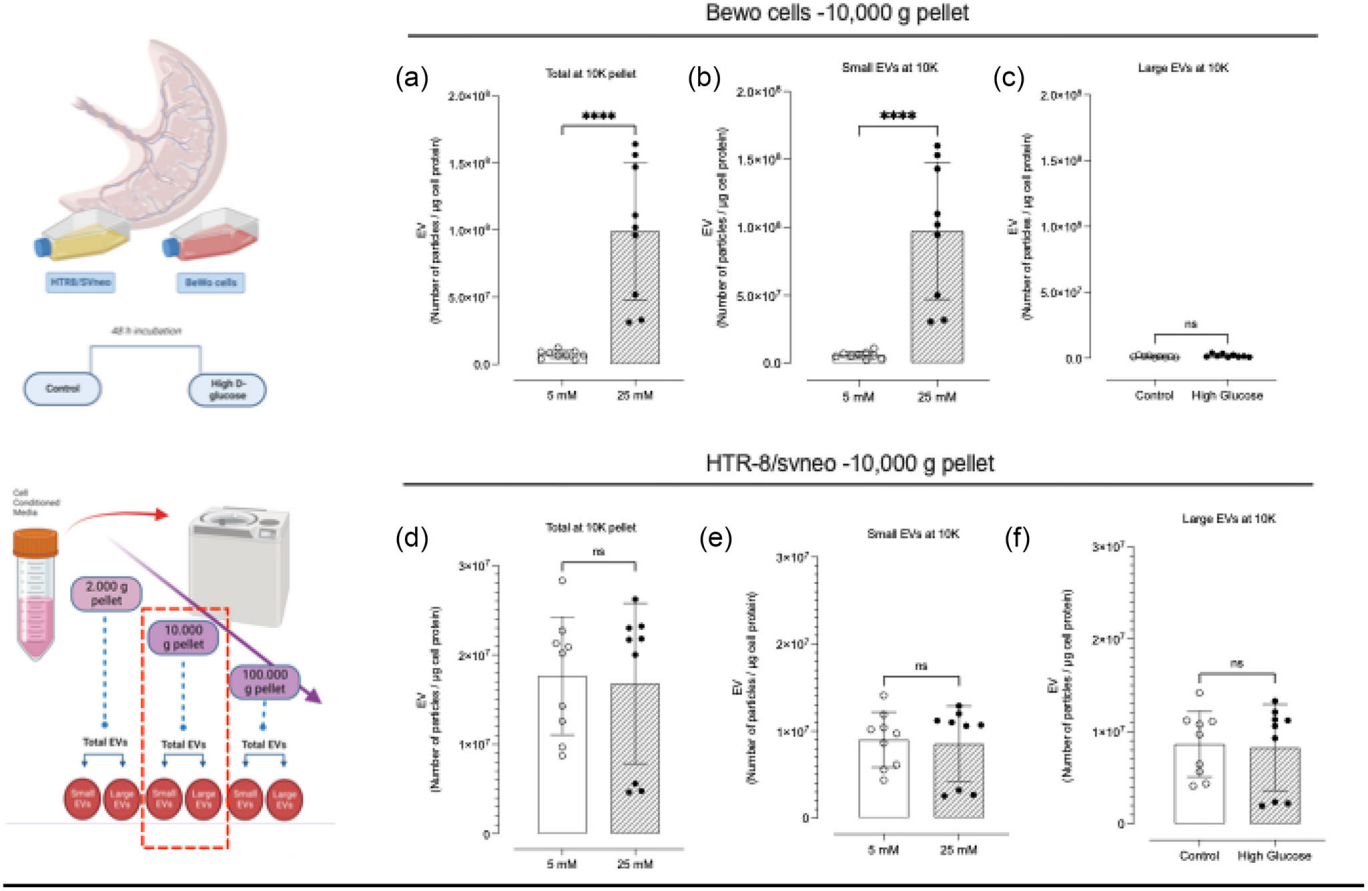
NTA of vesicles isolated at 10,000 g from BeWo cells and HTR8/SVneo. Total number of particles observed in samples isolated at 10,000 g after 40‐min centrifugation, and the respective analysis by size distribution categorised as small and large EVs. Samples considered were (a)–(c) *BeWo cell* and (d)–(f) *HTR8/SVneo*.

The assessment of the total number of particles contained in the 10K pellet obtained from BeWo cells showed an increase of ∼13.4‐fold (*p* < 0.0001) (Figure 3a), from 7.4 × 10^6^ particles per μg of cell protein in the control to 9.9 × 10^7^ particles per μg of cell protein in treated cells. The size mean and mode also changed significantly (Table 1). The mean decreased by 30.6%, going from 156.3 to 108.5 nm (*p* < 0.0001). The mode decreased from 109.8 to 94.5 nm (*p* = 0.0013). Further analysis showed that the variation observed is produced by the increase of small EVs (Figure 3b,c). The concentration of small EVs changed from 5.4 × 10^6^ to 9.5 × 10^7^ particles per μg of cell protein. Similar to the observations in the 2K pellet corresponding to HTR8/SVneo, no differences were observed in the total number of particles released or in specific sub‐populations (Figure 3d–f).

**FIGURE 4:**
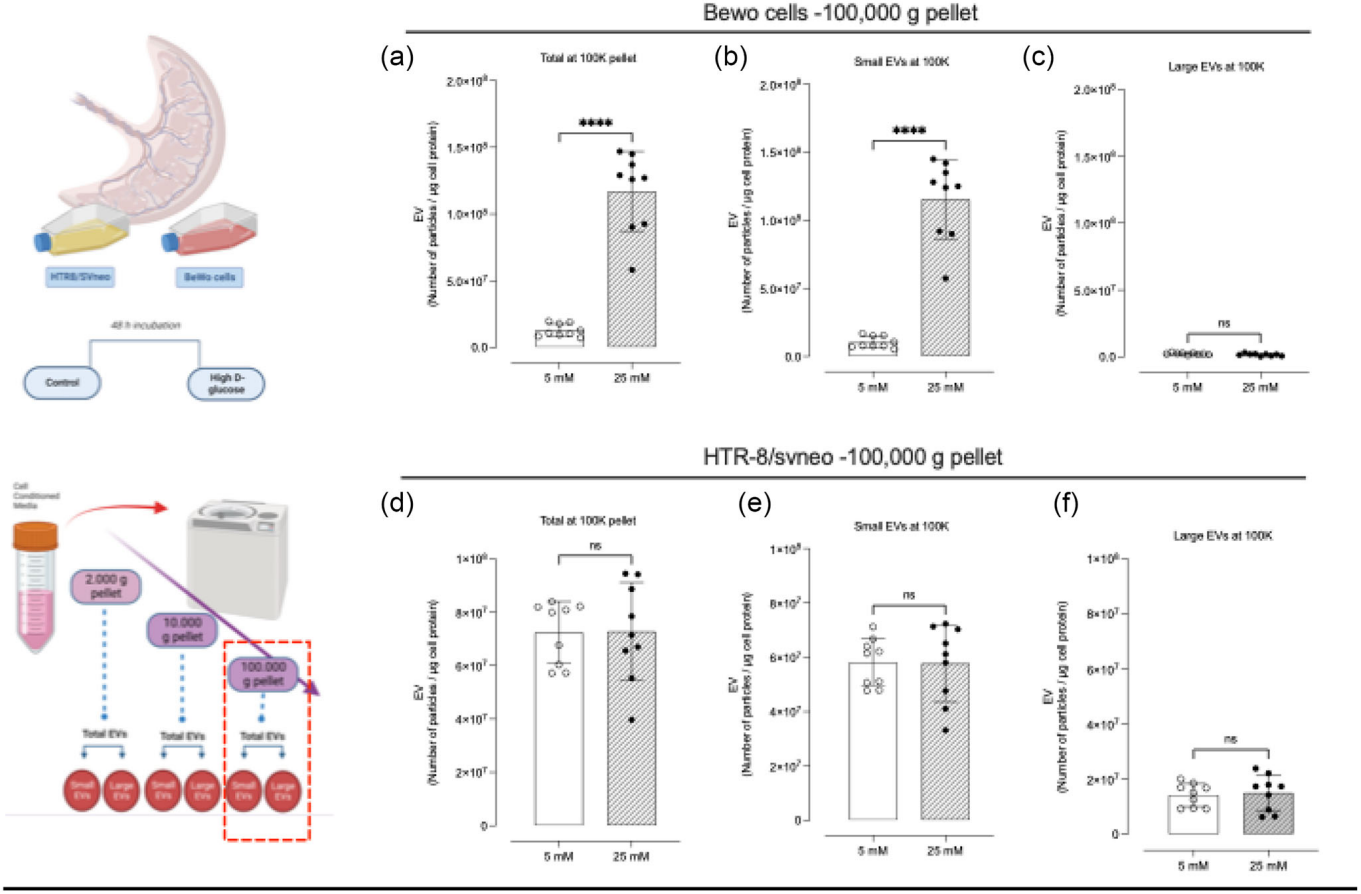
NTA of vesicles isolated at 100,000 g from BeWo cells and HTR/8/SVneo. Total number of particles observed in samples isolated at 100,000 g after 90‐min centrifugation, and the respective analysis by size distribution categorised as small and large EVs. Samples considered were (a)–(c) *BeWo cell* and (d)–(f) *HTR8/SVneo*.

Evaluation of the particles present in the 100K pellet exposed to high glucose showed ∼9.1‐fold higher (*p* < 0.0001) number of particles per μg of cell protein compared to the control (Figure 4a). The average size of the particles isolated from BeWo cells varied from 168.2 to 103.3 nm, which represented a reduction of 38.6% (*p* < 0.0001) (Table 1). On the other hand, the mode slightly decreased from 104.8 to 92.0 nm, which translated to a drop of 12.2%. Analysis of specific subpopulations affected by the treatment showed that small EVs were significantly increased in concentration (Figure 4b,c). Small EVs, a 12.8‐fold increment was observed from 8.9 × 10^6^ to 1.1 × 10^8^ particles per μg cell (*p* < 0.0001). In the case of HTR8/SVneo, there were no significant variations observed in the concentration of particles in a global context or a sub‐population level (Figure 4d–f). Analysis of size, mean and mode showed no differences between treatment and control (Table 1). This data established that placental cells from different origins respond differently to high glucose concentrations by altering the secretion of different populations of EVs. In this analysis, we used NTA to establish the differential concentration of particles isolated from the CCM obtained from placental cells. Next, we evaluate whether differences in the secretion of placenta cells derived EVs are associated with changes in the protein profile of placental cells.

### 3.3 Effect of high D‐glucose on the protein profile of placental cells

**FIGURE 5:**
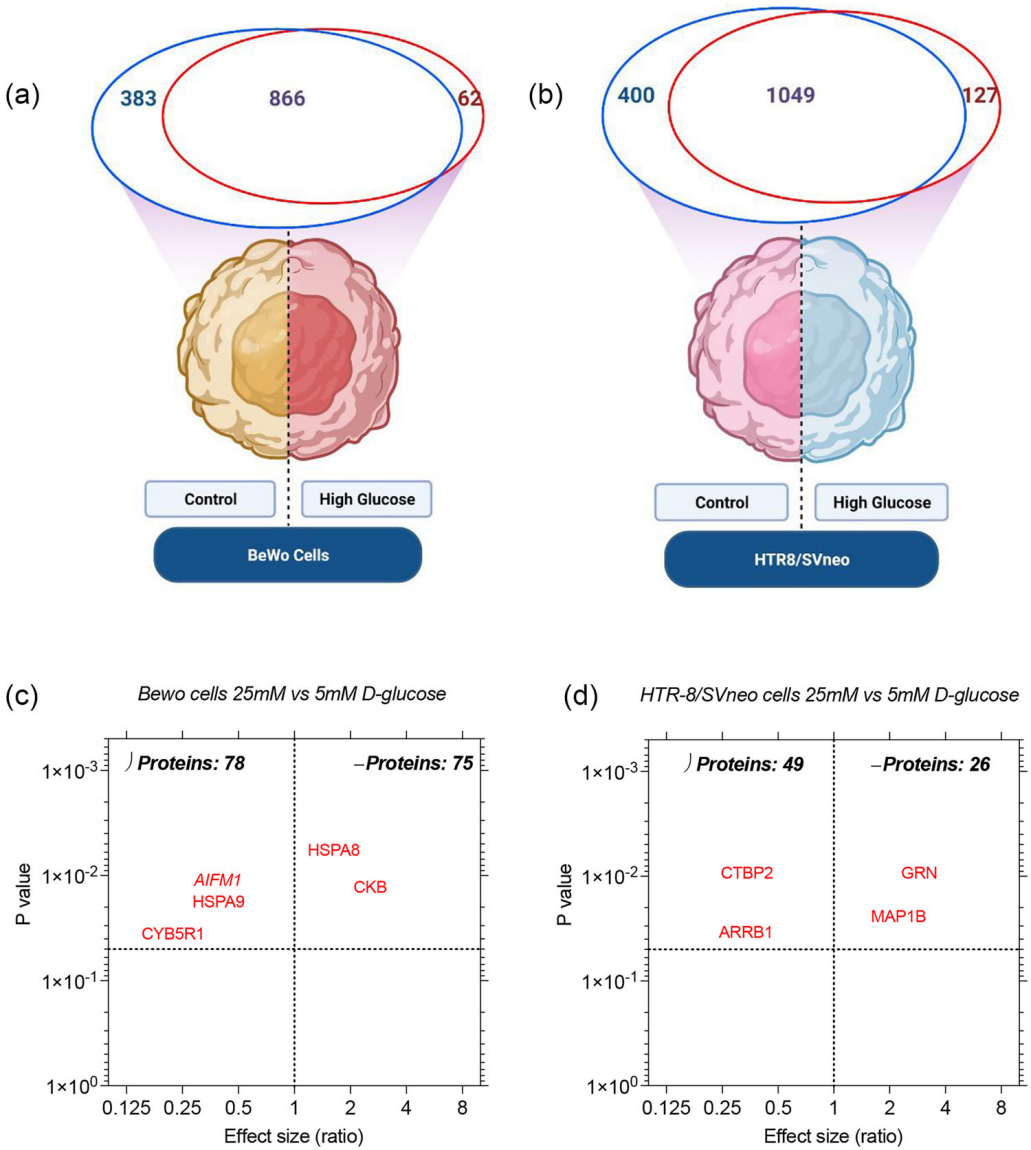
IDA results and volcano plot of SWATH results obtained in lysate from BeWo cell and HTR8/SVneo. The number of proteins identified by IDA analysis of lysate for (a) BeWo cells and (b) HTR8/SVneo. SWATH analysis was performed in both cell lines to determine the proteins that are upregulated or downregulated as a response to high glucose treatment. (c) Bewo cells, and (d) HTR8/SVneo. (a and b were created with BioRender.com).

A total of 1249 and 928 proteins were identified in BeWo cells under 5‐ and 25‐mM D‐glucose, respectively (Figure 5a). Common (866), and unique (383 at 5 mM, and 62 at 25 mM) proteins were identified in BeWo cells between the two groups. Similarly, a total of 1449 and 1176 proteins were identified in HTR8/SVneo cells under 5‐ and 25‐mM D‐glucose, respectively (Figure 5b). Common (1049), and unique (400 at 5 mM, and 127 at 25 mM) proteins were identified in HTR8/SVneo cells between the two groups. In order to identify variations in the levels of protein expression in these cell lines, quantitative proteomic analysis using SWATH was performed. The analysis settings for this comparison considered at least four peptides per protein with at least three transitions per peptide and FDR at 1%. In order to compare the different samples, iRT standards were used to align the results. We identified significant changes (*p* < 0.05) in a total of 155 proteins (77 and 78 down‐ and upregulated, respectively) in BeWo cells cultured under 25 mM compared to 5‐mM Dglucose (Figure 5c). HTR8/SVneo exhibited a different response and fewer proteins were significantly affected by high glucose treatment. A total of 49 proteins showed a significant decrease in expression after exposure to high glucose concentration. Conversely, 26 proteins increased in abundance after the 48‐h treatment (Figure 5d).

### 3.4 Bioinformatic analysis

The proteins identified using SWATH were further analysed using IPA. In favour of narrowing the search, a database that contains information related to proteins involved in vesicle trafficking was used. This analysis reported 24 proteins in BeWo cells (Supplementary Table 2) and 14 proteins in HTR8/SVneo (Supplementary Table 3). The proteins identified in BeWo cells were separated into four location categories. LGALS3 in the extracellular space, CDK2, CLIC3, RAN and SF3B3 in the nucleus, ITGB1, LAMP1, SLC2A1 and TLN1 in the plasma membrane, and finally, AIFM1, CKB, CYB5R1, GAPDH, HSPA8, HSPA9, HYOU1, IGF2BP1, LMAN2, NAE1, NAPA, RACK1, RHOA, S100A10 and SQSTM1 in the cytoplasm. For HTR8/SVneo, the proteins identified were grouped into three location categories. ERAP1 and GRN in the extracellular space, CTBP2, GAK, RUVBL2 and SART1 in the nucleus and ARRB1, HSPA5, MAP1B, MAPK3, NANS, PDCD6IP, RAB7A and UCHL1 in the cytoplasm. Interestingly, RAB7 has already been identified as a key regulator of the trafficking downstream of the multivesicular body, considerably affecting the release of small EVs. Furthermore, PDCD6IP (Alix) has been identified as a multifunctional protein involved in the biogenesis of EVs by the formation of the complex Syndecan/Syntenin/Alix. The proteins reported are not considered part of the conventional machinery related to EV biogenesis, trafficking/release and/or uptake. Taking that into consideration, a targeted mass spectrometry (MRM‐HR) protocol was used to analyse specific classical machinery.

### 3.5 Effect of high glucose on the classical machinery for biogenesis, trafficking/release and uptake of EVs

**FIGURE 6:**
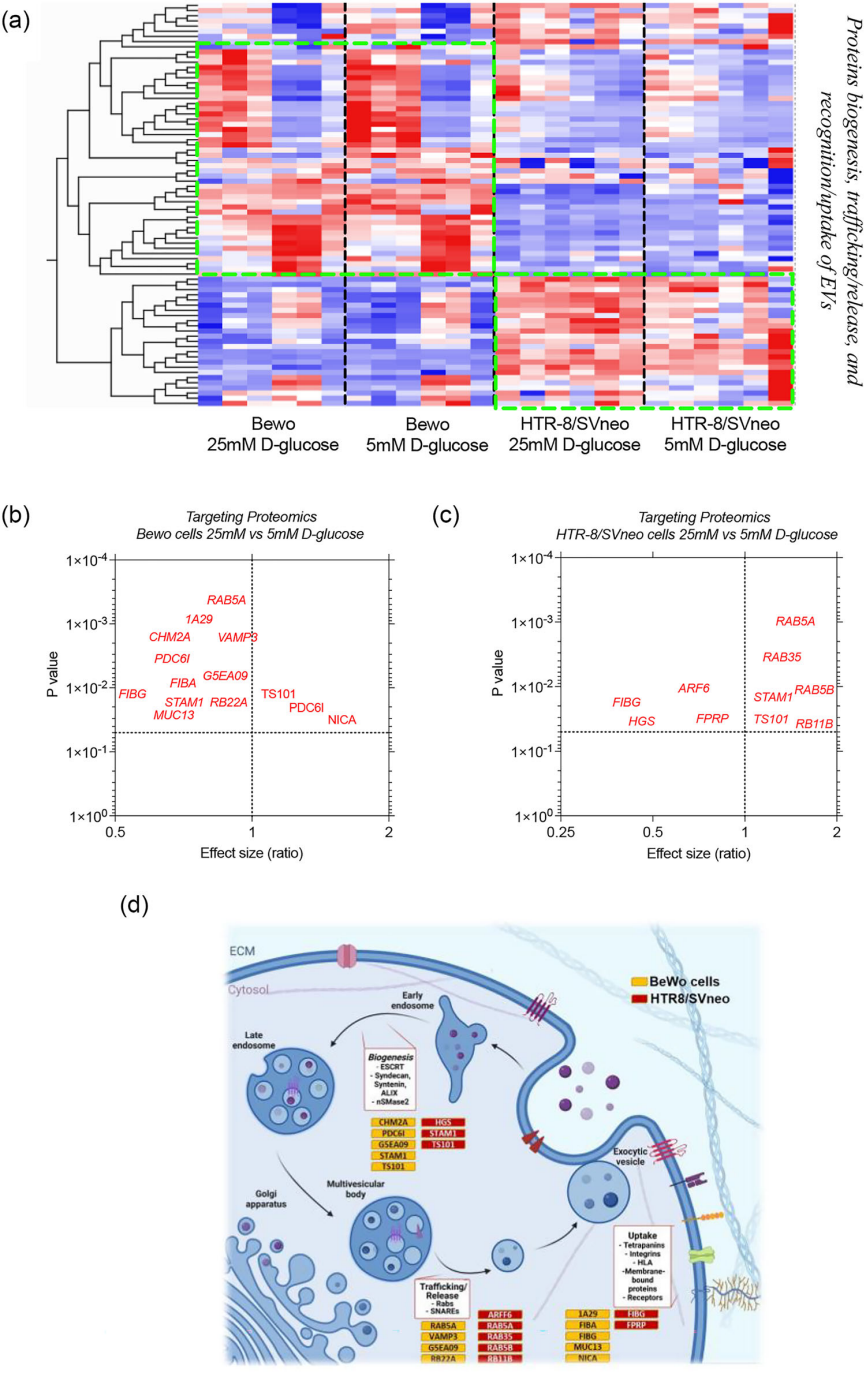
Targeting proteomic analysis for EV machinery in BeWo cells and HTR8/SVneo. (a) Heat map showing the specific analysis for proteins of the internal machinery in Bewo and HTR8/SVneo cultured at 5‐ or 25‐mM D‐glucose. Volcano plots showing the significant difference of proteins between cells culture at 5‐ versus 25‐mM D‐glucose in (b) Bewo cells and (c) HTR8/SVneo. (d) Representation of the proteins involved in EV biogenesis and their expression in Bewo and HTR8/SVneo cultured at 5‐ or 25‐mM D‐glucose. (d was created with BioRender.com).

The analysis of the mass spectrometry data established that high glucose changes the protein profile of BeWo cells, regulating proteins associated with the secretion and trafficking of EVs. Thus, we next designed a mass spectrometry protocol to target 37 well‐established proteins involved in the biogenesis, trafficking/release and recognition/uptake of EVs (Table 2). The initial evaluation contained 140 precursors in the protocol. Those precursors in which a signal was detected are presented in Figure 6. BeWo cells exposed to high glucose decreased TAS in 2/2 peptides evaluated in Rab‐5A, 1/2 peptides in HLA class I histocompatibility antigen, A‐29 alpha chain (HLA‐A), 1/3 peptides in Mucin‐13, 1/3 peptides in Prostaglandin F2 receptor negative regulator, 1/4 peptides in tumour susceptibility gene 101 protein, 1/3 peptides in STAM 1, 1/2 peptides in HLA class I histocompatibility antigen, A‐2 alpha chain (HLA‐A), 2/2 peptides in Nicastrin, 1/2 peptides in CD44, 1/2 peptides in Rab‐35, 1/3 peptides in Syntenin, 1/2 peptides in charged multivesicular body protein 4b, 1/2 peptides in Rab‐5B, 1/1 peptide in HLA class I histocompatibility antigen, C alpha chain, 2/2 peptides in charged multivesicular body protein 2a, 1/2 peptides in hepatocyte growth factor‐regulated tyrosine kinase substrate, 1/2 peptides in Rab‐7A, 1/2 peptides in Vacuolar protein sorting‐associated protein 28 homologue, 1/2 peptides in ADP‐ribosylation factor 6, 1/2 peptides in fibrinogen alpha chain and 1/2 peptides in Rab‐5C. This set of peptides exhibited the opposite trend in HTR8/SVneo which in general terms seems to increase TAS (Figure 6a).

**TABLE 2:** Peptides and the respective precursor evaluated by MRM‐HR for proteins related to EV machinery.

Protein accession	Protein description (alternative name)	Process	Peptides [position]	Precursor
O75886	Signal transducing adaptor molecule 2 (STAM2)	Biogenesis	K.VGSTPNGAK.D [35, 43]	415.7220++
			R.DFATEVR.A [87, 93]	419.2087++
			K.EDEDIAK.A [165, 171]	410.1902++
			K.QQHTETK.S [182, 188]	436.2170++
Q92783	Signal transducing adaptor molecule 1 (STAM1)	Biogenesis	K.NDPQLSLISAMIK.N [120, 132]	715.3896++
			K.DPGTVANK.K [162, 169]	401.2087++
			K.EEEDLAK.A [171, 177]	417.1980++
			K.LEDIDR.K [346, 351]	380.6954++
			K.HSELSELNVK.V [353, 362]	578.3039++
Q96CF2	Charged multivesicular body protein 4c (CHMP4c)	Biogenesis	R.AAPSPQEALVR.L [17, 27]	569.8144++
			K.QEYLENR.I [39, 45]	476.2302++
			K.QLTQIDGTLSTIEFQR.E [77, 92]	617.3267+++
			R.LPNVPSSSLPAQPNR.K [184, 198]	788.9257++
Q96H20	Vacuolar‐sorting protein SNF8 (VPS22)	Biogenesis	K.TNLEEFASK.H [44, 52]	519.7587++
			R.NGGLITLEELHQQVLK.G [114, 129]	598.0036+++
			K.FAQDVSQDDLIR.A [134, 145]	703.8492++
			R.QVLEHLLK.E [210, 217]	490.3004++
Q8NEZ2	Vacuolar protein sorting‐associated protein 37A (VPS37A)	Biogenesis	R.NSHSSIAEIQK.D [37, 47]	607.3122++
			K.IIQSLLDEFWK.N [114, 124]	696.3821++
			K.SIEELAR.K [274, 280]	409.2243++
			K.NLLLEPSLEAK.R [282, 292]	613.8532++
Q9UK41	Vacuolar protein sorting‐associated protein 28 homologue (H‐Vps28)	Biogenesis	K.YDNMAELFAVVK.T [35, 46]	700.3499++
			K.DCVSPSEYTAACSR.L [58, 71]	744.8083++
			R.LDCPLAMER.I [100, 108]	524.2517++
			K.EDRPITIK.D [111, 118]	486.2796++
			R.AMDEIQPDLR.E [148, 157]	594.2899++
			R.MSHLPPDFEGR.Q [166, 176]	643.3033++
Q99816	Tumour susceptibility gene 101 protein (TSG101)	Biogenesis	K.IYLPYLHEWK.H [108, 117]	681.3663++
			R.DGTISEDTIR.A [217, 226]	553.7698++
			R.ASLISAVSDK.L [227, 236]	495.7769++
			K.DEELSSALEK.M [294, 303]	560.7721++
Q9BY43	Charged multivesicular body protein 4a (CHMP4a)	Biogenesis	K.QEFLEQK.I [36, 42]	461.2374++
			K.IQQELQTAK.K [43, 51]	529.7957++
			R.FEQQLAQTDGTLSTLEFQR.E [71, 89]	738.0342+++
			R.EAIENATTNAEVLR.T [90, 103]	765.8916++
			K.LPSVPSTHLPAGPAPK.V [190, 205]	784.9434++
			K.VDEDEEALK.Q [206, 214]	524.2457++
G5EA09	Syndecan binding protein, isoform CRA_a (Syntenin‐1)	Biogenesis // trafficking/release	K.MSLYPSLEDLK.V [21, 31]	648.3312++
			R.DRPFER.T [211, 216]	410.2090++
			K.DSTGHVGFIFK.N [223, 233]	604.3089++
P18827	Syndecan‐1	Biogenesis // trafficking/release	K.EGEAVVLPEVEPGLTAR.E [100, 116]	883.4702++
			R.EQEATPRPR.E [117, 125]	542.2807++
			K.DEGSYSLEEPK.Q [281, 291]	627.2802++
			K.QANGGAYQKPTK.Q [292, 303]	631.8280++
Q8WUM4	Programmed cell death 6‐interacting protein (Alix)	Biogenesis	K.LALASLGYEK.S [110, 119]	532.8030++
			K.LANQAADYFGDAFK.Q [215, 228]	765.8648++
			K.EVFPVLAAK.H [239, 247]	487.2895++
			K.STPVNVPISQK.F [339, 349]	585.3299++
			K.ELPELLQR.N [438, 445]	499.2875++
O43633	Charged multivesicular body protein 2a (CHMP2a)	Biogenesis	R.ANIQAVSLK.I [80, 88]	472.2822++
			K.LPQIQK.I [118, 123]	363.7291++
			K.AEAAASALADADADLEER.L [197, 214]	596.9464+++
Q9H444	Charged multivesicular body protein 4b (CHMP4b)	Biogenesis	K.GGPTPQEAIQR.L [17, 27]	577.3016++
			K.IEQELTAAK.K [46, 54]	501.7769++
			K.QLAQIDGTLSTIEFQR.E [77, 92]	607.3231+++
			R.EALENANTNTEVLK.N [93, 106]	773.3914++
Q92542	Nicastrin	Recognition/uptake	K.IYIPLNK.T [39, 45]	430.7656++
			K.APDVTTLPR.N [314, 322]	485.2718++
			R.NQVEDLLATLEK.S [391, 402]	686.8696++
			K.ALADVATVLGR.A [485, 495]	543.3193++
			R.LLYGFLIK.A [520, 527]	483.8048++
P02675	Fibrinogen beta chain	Recognition/uptake	R.QDGSVDFGR.K [285, 293]	490.7252++
			K.QGFGNVATNTDGK.N [300, 312]	654.8126++
			K.IRPFFPQQ.‐ [483, 490]	516.7849++
P02671	Fibrinogen alpha chain	Recognition/uptake	K.NSLFEYQK.N [89, 96]	514.7560++
			R.GDFSSANNR.D [114, 122]	484.2150++
			R.LEVDIDIK.I [168, 175]	472.7686++
			K.VPPEWK.A [243, 248]	378.2080++
			K.VTSGSTTTTR.R [448, 457]	505.7593++
P02679	Fibrinogen gamma chain	Recognition/uptake	K.QSGLYFIKPLK.A [188, 198]	647.3819++
			R.LDGSVDFK.K [223, 230]	440.7242++
			K.NWIQYK.E [232, 237]	426.2241++
			R.VELEDWNGR.T [273, 281]	559.2673++
Q9H3R2	Mucin‐13	Recognition/uptake	K.CPDACNAQHK.Q [366, 375]	543.7264++
			R.STGFTNLGAEGSVFPK.V [470, 485]	806.4043++
			R.DSQMQNPYSR.H [493, 502]	613.2669++
			R.HSSMPRPDY.‐ [503, 511]	545.2427++
Q9P2B2	Prostaglandin F2 receptor negative regulator (CD9P‐1)	Recognition/uptake	R.EGEPFELR.C [160, 167]	488.7404++
			R.FHPGLGYEQR.Y [204, 213]	602.2989++
			K.APVLLSSLDR.K [741, 750]	535.8139++
			K.SPTGSWQK.E [800, 807]	445.7220++
P02786	Transferrin receptor protein 1	Recognition/uptake	R.LAGTESPVR.E [100, 108]	465.2562++
			R.LVYLVENPGGYVAYSK.A [208, 223]	886.4669++
				591.3137+++
			R.SSGLPNIPVQTISR.A [325, 338]	734.9095++
			R.DAWGPGAAK.S [409, 417]	436.7167++
			K.VSASPLLYTLIEK.T [495, 507]	717.4161++
P16070	CD44 antigen (ECMR‐III)	Recognition/uptake	R.FAGVFHVEK.N [29, 37]	517.2769++
			K.EQWFGNR.W [400, 406]	468.7198++
			R.TFIPVTSAK.T [571, 579]	482.2791++
			K.LVINSGNGAVEDR.K [681, 693]	672.3493++
			R.KPSGLNGEASK.S [694, 704]	544.2907++
P30508	HLA class I histocompatibility antigen, C alpha chain (HLA‐C)	Recognition/uptake	R.GYDQSAYDGK.D [135, 144]	552.2356++
			K.DYIALNEDLR.S [145, 154]	611.3091++
			R.EAEQWR.A [175, 180]	409.6932++
			K.WAAVVVPSGEEQR.Y [267, 279]	714.3675++
P01892	HLA class I histocompatibility antigen, A‐2 alpha chain (HLA‐A)	Recognition/uptake	R.FIAVGYVDDTQFVR.F [45, 58]	815.4172++
			R.FDSDAASQR.M [59, 67]	498.7227++
			R.GYYNQSEAGSHTVQR.M [106, 120]	566.2606+++
			K.WAAVVVPSGQEQR.Y [267, 279]	713.8755++
P30512	HLA class I histocompatibility antigen, A‐29 alpha chain (HLA‐A)	Recognition/uptake	R.FDSDAASQR.M [59, 67]	498.7227++
			K.AQSQTDR.A [92, 98]	403.1936++
			K.DYIALNEDLR.S [145, 154]	611.3091++
P62330	ADP‐ribosylation factor 6	Trafficking/release	K.FNVWDVGGQDK.I [58, 68]	632.8015++
			K.IRPLWR.H [69, 74]	420.7638++
			R.DAIILIFANK.Q [113, 122]	559.3344++
Q15286	Ras‐related protein Rab‐35	Trafficking/release	R.TVEINGEK.V [48, 55]	445.2349++
			K.LQIWDTAGQER.F [58, 68]	658.8333++
			R.TITSTYYR.G [71, 78]	502.7560++
			K.VVETEDAYK.F [128, 136]	527.2586++
Q9UL26	Ras‐related protein Rab‐22A (Rab‐22)	Trafficking/release	K.TVQYQNELHK.F [45, 54]	630.3226++
			K.EETFSTLK.N [89, 96]	477.7426++
			R.QHGPPNIVVAIAGNK.C [104, 118]	757.9255++
			R.IPSTDANLPSGGK.G [165, 177]	628.8277++
P59190	Ras‐related protein Rab‐15	Trafficking/release	K.TIEVDGIK.V [48, 55]	437.7477++
			R.AQGIFLVYDISSER.S [80, 93]	799.4147++
			R.EQGQQLAK.E [133, 140]	451.2405++
			R.LTELVLQAHR.K [164, 173]	590.3459++
Q15907	Ras‐related protein Rab‐11B (GTP‐binding protein YPT3)	Trafficking/release	K.NILTEIYR.I [166, 173]	511.2875++
P62491	Ras‐related protein Rab‐11A (YL8)	Trafficking/release	R.SIQVDGK.T [51, 57]	373.7058++
P51149	Ras‐related protein Rab‐7a	Trafficking/release	K.ATIGADFLTK.E [38, 47]	518.7873++
			R.DEFLIQASPR.D [103, 112]	588.3064++
			R.DPENFPFVVLGNK.I [113, 125]	738.3801++
			K.EAINVEQAFQTIAR.N [157, 170]	795.4177++
P51148	Ras‐related protein Rab‐5C (RAB5L)	Trafficking/release	K.NEPQNATGAPGR.N [184, 195]	606.2918++
			R.GVDLQENNPASR.S [198, 209]	650.3180++
P61020	Ras‐related protein Rab‐5B	Trafficking/release	K.SEPQNLGGAAGR.S [183, 194]	578.7889++
			R.GVDLHEQSQQNK.S [197, 208]	691.8366++
P20339	Ras‐related protein Rab‐5A	Trafficking/release	R.QASPNIVIALSGNK.A [120, 133]	706.3988++
			K.NEPQNPGANSAR.G [183, 194]	627.7947++
			R.GVDLTEPTQPTR.N [197, 208]	657.3384++
Q15836	Vesicle‐associated membrane protein 3 (VAMP‐3)	Trafficking/release	R.ADALQAGASQFETSAAK.L [49, 65]	833.4076++
				555.9408+++
P63027	Vesicle‐associated membrane protein 2 (VAMP‐2)	Trafficking/release	R.LQQTQAQVDEVVDIMR.V [31, 46]	624.9876+++
			R.ADALQAGASQFETSAAK.L [66, 82]	833.4076++
				555.9408+++
O14964	Hepatocyte growth factor‐regulated tyrosine kinase substrate (Hrs)	Biogenesis	R.QVEVNVR.N [98, 104]	422.2378++
			K.VEGHVFPEFK.E [136, 145]	594.8060++
			K.STYTSYPK.A [283, 290]	473.7295++
			R.LYYEGLQDK.L [464, 472]	564.7822++

However, peptides from the same protein showed differences after treatment. In order to obtain further evidence, these peptides were analysed according to how significantly they varied because of the treatment (Table 3). Using targeting proteomics, we targeted a total of 78 peptides representing 37 proteins, and identified 14 proteins (11 downregulated, and three upregulated in 25 mM D‐glucose) that significantly changed between BeWo cultured at 25 mM compared with 5 mM of D‐glucose (Figure 6b). Similarly, we identified 10 proteins (four downregulated, and six upregulated in 25‐mM D‐glucose) that significantly changed between HTR8/SVneo cultured at 25 mM compared with 5 mM of D‐glucose (Figure 6c). Proteins that changed in response to high glucose in BeWo included Syntenin‐1, charged multivesicular body protein 2a (CHMP2a), fibrinogen gamma chain (FIBG) and alpha chain (FIBA), Mucin‐13, and Ras‐related protein Rab‐5A and RB22A, Nicastrin (NICA), tumour susceptibility gene 101 protein (TSG101), signal transducing adaptor molecule 1 (STAM1), vesicle‐associated membrane protein 3 (VAMP‐3) and programmed cell death 6‐interacting protein (Alix). In the case of HTR8/SVneo, peptides that decreased belong to FIBG, ADP‐ribosylation factor 6 (ARF6), hepatocyte growth factor‐regulated tyrosine kinase substrate (HGS), prostaglandin F2α receptor regulatory protein (FPRP), RAB5A, RAB35, RAB5B, and RB11B, STAM1 and TSG101. These data provide evidence that high glucose changes the expression of classical proteins associated with the biogenesis, trafficking/release and recognition/uptake of EVs, in placental cells, which could explain in part changes in the concentration of circulating EVs in diabetic pregnancies (Figure 6d).

**TABLE 3:** Summary MRM‐HR results at peptide level for BeWo cells and HTR8/SVneo.

Protein accession	Protein description (Alternative name)	Peptides [position]	Precursor	Cell line	Change	Fold change	*p*‐value
G5EA09	Syndecan binding protein, isoform CRA_a (Syntenin‐1)	K.DSTGHVGFIFK.N [223, 233]	604.3089++	BeWo	↓	0.83	0.004048
O43633	Charged multivesicular body protein 2a (CHMP2a)	R.ANIQAVSLK.I [80, 88]	472.2822++	BeWo	↓	0.82	0.039344
P02679	Fibrinogen gamma chain	R.LDGSVDFK.K [223, 230]	440.7242++	BeWo	↓	0.81	0.027414
Q9H3R2	Mucin‐13	R.HSSMPRPDY.‐ [503, 511]	545.2427++	BeWo	↓	0.80	0.003643
P20339	Ras‐related protein Rab‐5A	R.QASPNIVIALSGNK.A [120, 133]	706.3988++	BeWo	↓	0.76	0.003529
Q92783	Signal transducing adaptor molecule 1 (STAM1)	K.HSELSELNVK.V [353, 362]	578.3039++	HTR8/SVneo	↑	1.24	0.000272
Q99816	Tumour susceptibility gene 101 protein (TSG101)	K.DEELSSALEK.M [294, 303]	560.7721++	HTR8/SVneo	↑	1.32	0.021099
Q9P2B2	Prostaglandin F2 receptor negative regulator (CD9P‐1)	R.FHPGLGYEQR.Y [204, 213]	602.2989++	HTR8/SVneo	↑	1.41	0.004456
		K.SPTGSWQK.E [800, 807]	445.7220++	HTR8/SVneo	↓	0.69	0.020449
P62330	ADP‐ribosylation factor 6	K.IRPLWR.H [69, 74]	420.7638++	HTR8/SVneo	↓	0.33	0.037714
Q15907	Ras‐related protein Rab‐11B (GTP‐binding protein YPT3)	K.NILTEIYR.I [166, 173]	511.2875++	HTR8/SVneo	↑	1.29	0.004723
P61020	Ras‐related protein Rab‐5B	K.SEPQNLGGAAGR.S [183, 194]	578.7889++	HTR8/SVneo	↑	1.23	0.028461
O14964	Hepatocyte growth factor‐regulated tyrosine kinase substrate (Hrs)	R.QVEVNVR.N [98, 104]	422.2378++	HTR8/SVneo	↓	0.57	0.045210

### 3.6 Effect of proteins associated with EV biogenesis on cell response to high glucose

In our previous results, it was observed that Bewo and HTR‐8 cells exhibited differential responses to glucose in terms of EV secretion. Specifically, high glucose levels led to an increase in the secretion of small EVs from Bewo cells, but this effect was not observed in HTR8/SVneo cells cultured under high glucose conditions. Furthermore, our quantitative proteomics analysis identified a set of proteins associated with EV biogenesis, trafficking/release and uptake that could potentially account for the differential responses of placental cells to high glucose.

To assess the effect of specific proteins (i.e. HSPA8, ERAP1, HSPA9, RAB7A, PDCD6IP, SDCBP, RAB5A, STAM, RAB11B and RAB5B) on the cellular response to high glucose, we transfected protein overexpression vectors into HEK293T cells and exposed them to either 5‐ or 25‐mM D‐glucose (Figure S2 and Table 1). Western blot analysis confirmed significantly higher protein expression in cells transfected with the overexpression vectors compared to control cells (see Figure S3).

**FIGURE 7:**
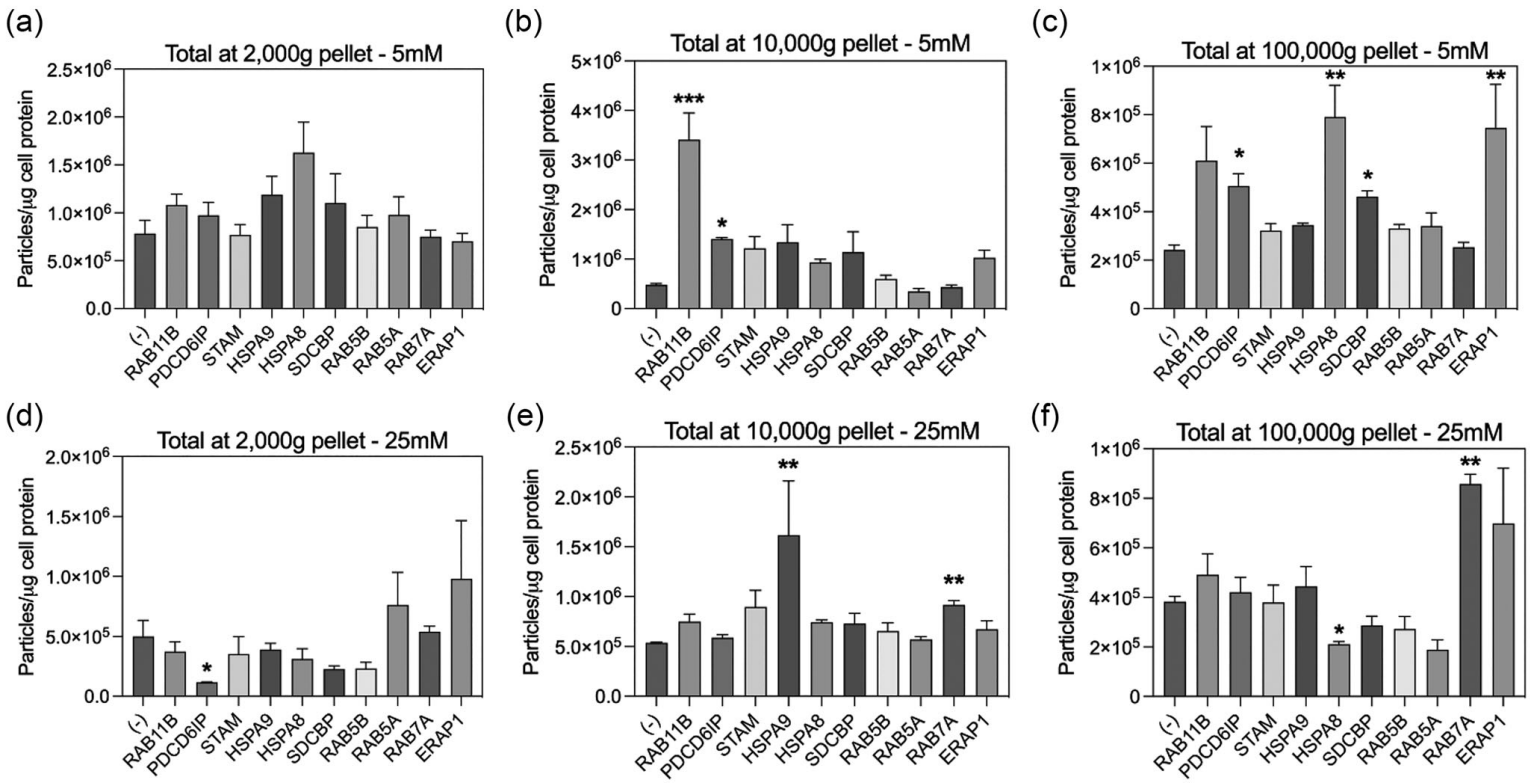
NTA of vesicles isolated from HEK293T cells overexpressed with candidate proteins. Plasmid vectors with the candidate proteins (RAB11B, PDCD6IP, STAM, HSPA9, HSPA8, SDCBP, RAB5B, RAB5A, RAB7A, ERAP1) and empty vector as control (‐) was overexpressed in HEK293T cells and exposed to low (5 mM) and high glucose (25 mM). The cell conditioned media was collected and EVs were isolated by differential centrifugation followed by ultracentrifugation. EV pellet at 2000, 10,000 and 100,000 g was analysed by NTA. (a) 2000‐g pellet from cells cultured at 5‐mM glucose. (b) 10,000‐g pellet from cells cultured at 5‐mM glucose. (c) 100,000‐g pellet from cells cultured at 5‐mM glucose. (d) 2000‐g pellet from cells cultured at 25‐mM glucose. (e) 10,000‐g pellet from cells cultured at 25‐mM glucose. (f) 100,000‐g pellet from cells cultured at 25‐mM glucose. In (a), Kruskal–Wallis test *p* > 0.05. In (b)–(f), Kruskal–Wallis test, *p* < 0.05, and Dunn's multiple comparison test versus control (‐) *p < 0.05, **p < 0.005 and ***p < 0.0005.

To evaluate the impact of high glucose on the secretion of EV from transfected HEK293T cells, we used NTA to determine the size and concentration of EVs in each pellet. Overexpression of specific proteins altered the concentration of EV released from cells at high and low glucose (Figure 7a–f). On analysis of EV concentrations in the 2000‐g pellet, we identified that cells transfected with PDCD6IP overexpression vector decreased the secretion of EV (defined as particles per microgram cell protein at 24 h) compared to control cells when cells were incubated with 25‐mM glucose. Interestingly, no effect of overexpression vectors was observed when cells were incubated with 5‐mM glucose (Figure 7a,d).

Evaluation of 10,000‐g pellet revealed that cells transfected with RAB11B, and PDCD6IP overexpression vectors increased the release of EVs compared with control cells at 5‐mM glucose. In contrast HSPA9 overexpression caused an increase in EV release compared to control cells at 25 mM (Figure 7b,e).

At 100,000‐g pellet, cells transfected with PDCD6IP, HSPA8, SDCBP and ERAP1 overexpression vectors increased the release of EVs at 5‐mM glucose compared to control cells (Figure 7c). Moreover, cells transfected with HSPA8 and RAB7A overexpression vectors at 25‐mM glucose increase the secretion of EV compared to control cells (Figure 7f). Interestingly, analysis of the sub‐population of EV classified by size revealed that the effect of cells transfected with overexpression vectors mainly regulated the secretion of small EV (Supplemental material 4).

This comprehensive analysis sheds light on the complex interplay between protein expression and glucose levels, contributing to our understanding of how these factors impact the secretion of EVs. Interestingly, we observed a significant increase of EV in the 10 and 100K pellet from cells transfected with RAB7A vector and incubated with 25‐mM glucose (2‐fold and 3.5‐fold increase in 10 and 100K compared to low glucose and control cells, respectively) (Figure S5), making this protein and attractive target to evaluate his expression in placental tissue obtained from GDM pregnancies.

### 3.7 RAB7A‐related changes in placental tissues of women with GDM

**FIGURE 8:**
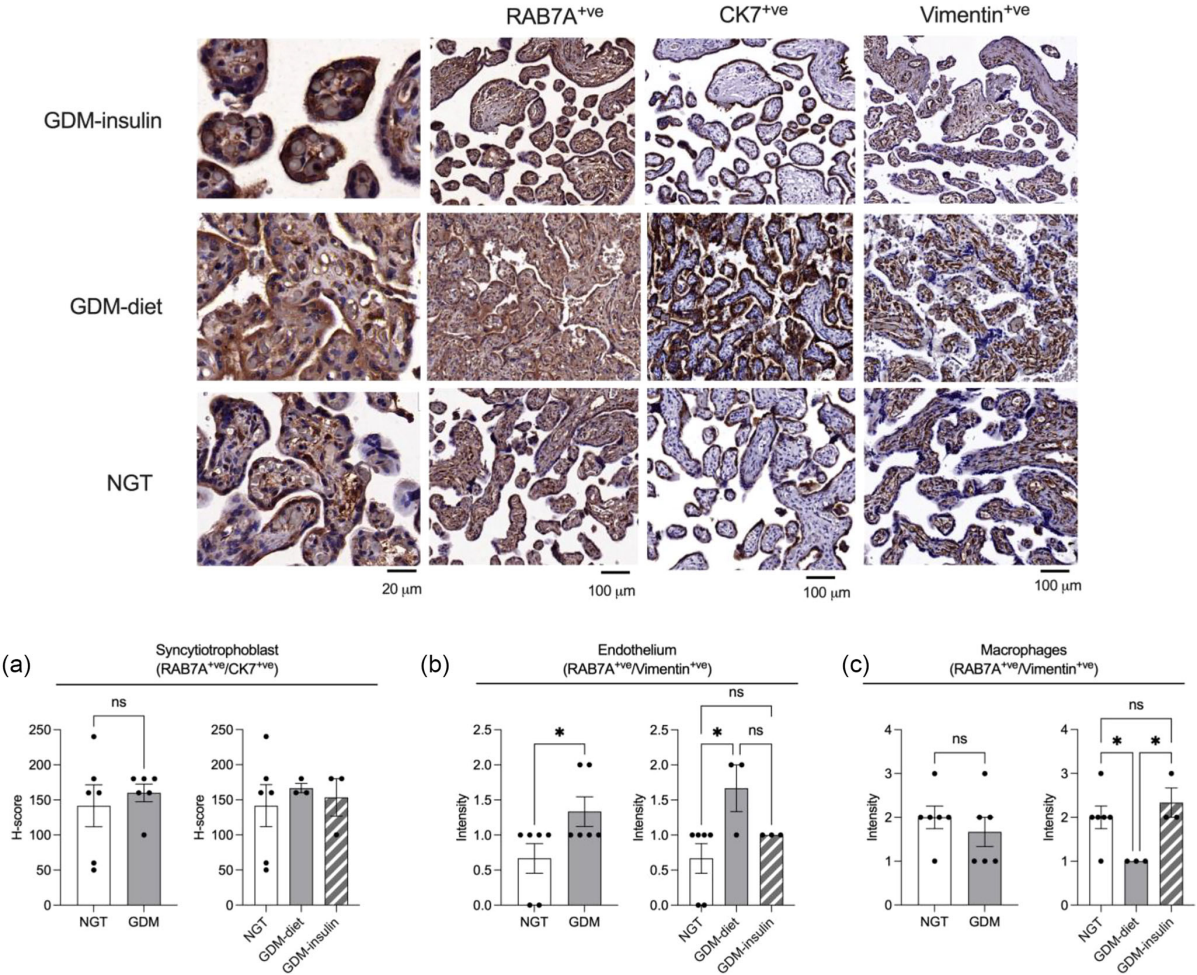
Immunohistochemistry of placental tissue from NGT, GDM‐diet and GDM‐insulin patients. (a) Immunostaining of formalin‐fixed and paraffin‐embedded placental tissue from GDM‐insulin, GDM‐diet and NGT patients with RAB7A, CK7 and vimentin antibodies. *H*‐score for RAB7A staining in (b) CK7 positive syncytiotrophoblasts, (c) vimentin positive endothelial cells, (d) vimentin positive Hofbauer cells.

Given the increasing body of evidence suggesting that RAB7A plays a crucial role in EVs (mainly exosome) biogenesis and secretion as a key regulatory protein within the endosomal pathway (Baietti et al., 2012), we conducted immunohistochemical staining of placental tissues. Demographic data of all participants involved in this study are summarized in Table 4. Histological examination of the placental sections from women with NGT test, diet treated GDM and insulin treated GDM was performed after staining with anti‐ RAB7A, anti‐CK7 and anti‐vimentin antibodies (Figure 8a). The CK7 immunostaining was localized in the syncytiotrophoblastic layer and vimentin was localized in the stromal cells and endothelial cells of placental villi. RAB7A showed similar immunostaining intensity in CK7 and vimentin positive cells. However, the proportion of cells stained with RAB7A was lesser among the vimentin positive cells compared to CK7 positive cells. The staining characteristics were similar for NGT and GDM patients.

**TABLE 4:** Clinical characteristics of patients.

	NGT (*n* = 6)	GDM‐diet (*n* = 3)	GDM‐insulin (*n* = 3)
**Maternal characteristics**			
Age (years)	28 ± 6.512 (21.75, 34.25)	26 ± 2 (24, 28)	34.33 ± 4.041 (30, 38)
Height (cm)	164.3 ± 7.711 (156.8, 171)	160.7 ± 3.786 (158, 165)	158.3 ± 6.028 (152, 164)
Pre‐pregnancy weight (kg)	79.95 ± 25.44 (63.5, 89.5)	71 ± 22.34 (53, 96)	71.33 ± 31.01 (40, 102)
Pre‐pregnancy BMI (kg/m^2^)	29.45 ± 8.167 (23.95, 33.6)	27.3 ± 7.3 (21, 35.3)	28.13 ± 11.56 (17.3, 40.3)
Weight at delivery (kg)	81.6 ± 5.595 (76.5, 86.5)	78.67 ± 22.5 (61, 104)	75 ± 28.62 (45, 102)
BMI at delivery (kg/m^2^)	30.82 ± 3.701 (27.5, 34.5)	30.23 ± 7.227 (24.1, 38.2)	29.6 ± 10.41 (19.5, 40.3)
Gestational age (weeks)	39.2 ± 1.307 (38.2, 39.93)	38.8 ± 0.7211 (38.2, 39.6)	38.8 ± 0.6245 (38.3, 39.5)
Gestational age (days)	276 ± 9.187 (268.8, 281.5)	273 ± 5.568 (268, 279)	273 ± 4.583 (269, 278)
Gravida	1.667 ± 0.8165 (1, 2.25)	2 ± 1 (1, 3)	4.333 ± 3.215 (2, 8)
Parity	1.667 ± 0.8165 (1, 2.25)	1.667 ± 1.155 (1, 3)	2.333 ± 0.5774 (2, 3)
**Maternal OGTT at ∼28** **weeks gestation**			
Fasting plasma glucose (mmol/L)	4.167 ± 0.1528 (4, 4.3)	4.833 ± 0.6429 (4.1, 5.3)	4.25 ± 0.07071 (4.2, 4.3)
1‐h plasma glucose (mmol/L)	7.8 ± 0.4967 (7.425, 8.325)	10.07 ± 1.159^*^ (9, 11.3)	9.3 ± 0.4243 (9, 9.6)
2‐h plasma glucose (mmol/L)	6.2 ± 1.217 (4.8, 7)	8.733 ± 0.6506^*^ (8.1, 9.4)	8.3 ± 0.2828 (8.1, 8.5)
**Foetal characteristics**			
Placental weight (g)	684.5 ± 143.4 (537.5, 787)	695 ± 63.64 (650, 740)	585.5 ± 232.6 (421, 750)
Foetal sex (male/female)	3/3	1/2	1/2
Birth weight (g)	3543 ± 321.8 (3210, 3815)	3510 ± 280.5 (3240, 3800)	3103 ± 83.86 (3050, 3200)
Foetal length (cm)	50.25 ± 1.605 (48.75, 51.38)	49.83 ± 0.2887^*^ (49.5, 50)	50.83 ± 0.2887^ǂ^ (50.5, 51)
Ponderal index (g/cm^3^)	2.807 ± 0.3393 (2.665, 3.008)	2.833 ± 0.1888 (2.67, 3.04)	2.363 ± 0.1012 (2.3, 2.48)
Apgar5'	9.5 ± 0.5477 (9, 10)	9 ± 0 (9, 9)	9.333 ± 0.5774 (9, 10)

Values represent mean ± SD (inter‐quartile range). Differences in variables were compared using one‐way ANOVA test.

Abbreviation: BMI, body mass index.

* *p* < 0.05 NGT versus GDM‐diet. ǂ*p* < 0.05 NGT versus GDM‐insulin.

Further, *H*‐score analysis indicates no significant difference in RAB7A expression in the syncytiotrophoblast layer of placenta between NGT and GDM patients (Figure 8a). Moreover, when we categorized the data by treatment, no significant differences in RAB7A expression in syncytiotrophoblasts were noted between GDM individuals treated with diet or insulin compared to NGT individuals. However, in the endothelial cells of placental villi, there was a notably higher expression of RAB7A in the GDM group compared to the NGT (Figure 8b), with these differences being particularly pronounced in placental tissues from GDM individuals treated with diet. Intriguingly, RAB7A expression in Hofbauer cells was significantly lower in GDM individuals treated with diet compared to those treated with insulin and NGT individuals (Figure 8c). Our immunohistochemical analysis of RAB7A revealed distinct staining patterns in placental tissues from individuals with NGT and GDM.

## 4 DISCUSSION

EVs have been shown to have a key role in maternal–foetal communication (Nakahara et al., 2020), including in pregnancies associated with hyperglycaemia and GDM (Palma et al., 2022). However, little is known about how placental cells can modify the secretion of EVs into the maternal circulation, in response to the extracellular milieu. The principal findings of this study were (i) high glucose regulates the secretion of different populations of EVs (quantified by NTA) from placental cells (evaluated using trophoblast cell lines HTR8/SVneo and BeWo), (ii) using a state‐of‐the‐art proteomic quantification approach, we were able to identify specific changes in the protein profile of placental cells in response to high glucose. The changes in the secretion profile of EVs in response to high glucose were associated with the modification of proteins involved in the trafficking of multivesicular bodies to the plasma membrane, (iii) RAB7A in placental cells might be involved in differential secretion of EVs in response to changes in extracellular glucose levels. The results in placental cells reported herein might serve as the foundation for the discovery of potential therapeutic targets for obstetric disorders associated with hyperglycaemia, such as GDM.

Primary trophoblast cells are the most suitable models representing normal trophoblast behaviour and physiology. Due to the unavailability of freshly isolated primary placental cells for this study, we have used trophoblast cell lines HTR8/SVneo and BeWo cells to evaluate the effect of high glucose or diabetic environment on placental EV release. Even though HTR8/SVneo and BeWo cells are widely used as placental models, the limitations of their use need to be addressed. BeWo cells correspond to choriocarcinoma cells with properties related to villous trophoblast (Pattillo & Gey, 1968). This cell line has a fusogenic nature that can be triggered by treatment with forskolin, inducing syncytialisation, leading to the formation of the syncytiotrophoblast (Al‐Nasiry et al., 2006). Because of its characteristics, it has been widely used in studies of syncytialisation processes, hormone secretion and as in vitro placental barrier model (Abbas et al., 2020; Ramos et al., 2008; Yashwanth et al., 2006). However, BeWo cells are cancer derived and hence might not fully represent normal trophoblast physiology. On the other hand, HTR8/SVneo is a first‐trimester extravillous trophoblast commonly used to study extravillous trophoblast invasion and proliferation (Graham et al., 1993). This cell line contains both trophoblasts and stromal cells and is considered a good model to evaluate the epithelial‐to‐mesenchymal transition (Abou‐Kheir et al., 2017; Msheik et al., 2020). Considering these factors, results from these cell lines need to be interpreted in the context of these limitations, before they can be extrapolated to explain the development of complications during pregnancy.

The proper development of the placenta allows the establishment of a maternal–foetal communication system that will support the development of the baby and is crucial for a healthy pregnancy (Cindrova‐Davies & Sferruzzi‐Perri, 2022). Maternal–foetal communication is essential for a successful pregnancy, and multiple circulating factors or extracellular cues can alter the function of the placenta leading to changes in maternal physiology. Maternal hyperglycaemia has been related to adverse outcomes in pregnancy (Catalano et al., 2012; Yogev et al., 2010), and is associated with changes in the EV levels and function throughout gestation (Salomon et al., 2014; Sarker et al., 2014). In this study, EVs from HTR8/SVneo and BeWo cells treated with high concentrations of glucose (to mimic the maternal diabetic environment) were first separated into different sizes by differential centrifugation followed by ultracentrifugation and characterized by presence of markers associated with EVs (e.g. CD9, and TSG101). The placental origin of these EVs was established using the presence of a placental marker, PLAP. PLAP was identified across all pellets (i.e. 2K, 10K and 100K) suggesting that PLAP is a marker present across all types of EVs. The tetraspanin protein CD9 was significantly higher in the 100K pellet compared to the 2 and 10K pellets, in correlation with an enrichment of small EVs such as exosomes in the 100K pellet. Interestingly, we identified a differential response to high glucose in BeWo compared with HTR8/SVneo cells. BeWo cells in the presence of high glucose secreted higher levels of EVs to the extracellular milieu across all pellets studied. Whereas, HTR8/SVneo cells showed no changes in the concentration of EVs in CCM.

Alterations in the levels of EV release in the presence of high glucose has been reported with different cell types (Sáez et al., 2018; Zhu et al., 2019). For instance, human umbilical vein endothelial cells (HUVECs) exposed to 25‐mM D‐glucose increased the release of EVs compared to normal glucose concentration (Sáez et al., 2018). Similarly, macrophages exposed to 30‐mM D‐glucose increased the number of EVs secreted into the media compared to cells cultured with 5‐mM glucose. Additionally, high glucose induced changes in the morphology and function of macrophages (Zhu et al., 2019). The reason for the differential effect of high glucose on EV release from HTR8/SVneo and BeWo cells in this study and its association to the physiological response of these cells in pregnancy is not clear and requires future investigations.

We used differential centrifugation followed by ultracentrifugation for EV isolation. Ultracentrifugation might have yielded EV preparations with lower purity as compared to other methods of EV isolation such as size exclusion chromatography and density gradient centrifugation (Lobb et al., 2015), but is the most time‐efficient method for processing large numbers of samples. Further, we used NTA for sizing the EVs released from cells. The precision of NTA can vary depending on several factors, including instrument quality, sample quality and data analysis techniques. NTA is generally known for its ability to provide precise size and concentration measurements of nanoparticles within its specified size range, typically from around 30 nm to 1 μm (Bachurski et al., 2019; Comfort et al., 2021; Thane et al., 2019). Previous studies have established the reproducibility of the NTA with a coefficient of variation (CV) range: 5.4%–10.7%, and 0.8%–6.7% for concentration and size, respectively (Bachurski et al., 2019). There are several techniques to enumerate and characterize EVs including tunable resistive pulse sensing (tRPS), Single particle interferometric reflectance imaging sensor (SP‐IRIS) and high‐resolution flow cytometry (Thery et al., 2018). However, the accuracy of particle sizing can be affected with various bias associated with the technique and the concentration and size range of the particles might vary with the platform (Thery et al., 2018). The limitations of NTA include variability with light scattering properties of complex samples (Gardiner et al., 2013) and with instrument settings (Maas et al., 2015) and also, overestimation of EV numbers as it not specific to EVs (Thery et al., 2018).

In our study, the changes in the release of EVs in response to high glucose from BeWo cells are primarily driven by small EVs (<200 nm). Small EVs have been extensively studied for their mode of origin/biogenesis, cargo sorting and expression of membrane markers, but also for their regulatory roles in several cellular mechanisms/functions (Colombo et al., 2013; Fader et al., 2009; Henne et al., 2011; Kooijman et al., 2003; Mulcahy et al., 2014; Piper & Luzio, 2007; Yuyama et al., 2012). Extracellular factors including pH, glucose, oxygen concentration and thermal shocks can modulate the release, content and bioactivity of EVs (Borges et al., 2013; Clayton et al., 2005; da Silva Novaes et al., 2019; Logozzi et al., 2018; Rice et al., 2015). In order to elucidate the mechanism of differential EV release in response to high glucose, we performed proteomic characterization in cells followed by a targeted mass spectrometric approach to identify specific proteins involved in the biogenesis of small EVs (predominantly exosomes). We identified that a set of proteins that form nearly 66% of the cell proteome was invariable between control and treatment with high D‐glucose groups (Figure 5a), with both cell lines and represents the proteome associated with the basic and conserved biological processes of the cell.

D‐glucose treatment had a significant effect on BeWo cells by affecting proteins that have been shown to be involved (although in other models/studies) in apoptosis, angiogenesis, proliferation and cancer among many other processes (Tables 2 and 3). Several of these proteins could be related to the choriocarcinoma origin of BeWo cells, however, it needs to be considered that the results obtained by IPA correspond to those significantly altered by the treatment and whilst they can be related to cancer, the biological functions of these proteins are not unique to cancer itself. The same response was observed in HTR8/SVneo, where apoptosis, proliferation, cell growth and unfolded protein response were among several biological functions affected by the proteins modified by high D‐glucose exposure.

In relation to the machinery behind small EV dynamics (including biogenesis and trafficking), we identified differential expression of proteins associated with EV biogenesis, trafficking/release and uptake. Interestingly, several proteins present in the exosome biogenesis pathway (RAB5A, STAM1, VAMP3) were downregulated in BeWo cells exposed to high glucose, whereas as upregulated (RAB5A, RAB5B, STAM1, TSG101) in HTR8/SVneo cells at high glucose. This could be explained partly by the fact that these proteins are exported from the cells as cargo in EVs causing a decrease in their abundance in high EV releasing BeWo cells. Also, it is necessary to consider that the protocol used here (MRM^HR^) needs to be corroborated with standard proteins or peptides. Future potential strategies for absolute quantification of these specific proteins (classical machinery) in order to fully understand the effect of high glucose on the secretion of different types of EVs need to be considered, including the use of protein concatenates, synthetic proteins or peptides but also their protein microenvironment (which is not the same as cellular microenvironment).

Further, to understand the role of proteins in the regulation of EV release in response to extracellular glucose concentration, we chose 10 candidate proteins (RAB11B, PDCD6IP, STAM, HSPA9, HSPA8, SDCBP, RAB5B, RAB5A, RAB7A and ERAP1) from our proteomic analysis. The effect of these proteins on EV release in the presence of high and low glucose was identified by overexpression analysis. We identified that these proteins regulate the release small and large EVs in a specific pattern in response to low and high glucose. For example, protein PDCD6IP increased the concentration of EVs in 2 and 10K pellet at low glucose whereas did not significantly affect EV concentrations in 100K pellet. On the other hand, RAB7A increased the concentration of EVs in 10 and 100K pellet in response to high glucose. Rab protein family has been extensively studied in relation to intracellular dynamics in areas such as protein recycling, immunology and EV biogenesis (Baietti et al., 2012; Lapierre et al., 2003; Savina et al., 2005; Savina et al., 2002; Schlierf et al., 2000; Wang & al. et al., 2007). Evidence has identified Rab proteins as key elements in biogenesis and trafficking of EVs (Colombo et al., 2014; Nagano et al., 2019; Savina et al., 2005; Savina et al., 2002; Schlierf et al., 2000). However, this family encompasses several members, and they are specifically regulated according to cell type and stimulus. For example, it is known that Rab11a knocked‐down K562 cells exhibit a decrease in exosome release and trafficking/recycle of transferrin receptor (Savina et al., 2002). Rab7B, is a member known by its role in the transport and degradation of proteins in endosomes and lysosomes (Wang & al. et al., 2007) and Rab5A is critical player in endosome maturation by recruitment of Rab7A (Huotari & Helenius, 2011). In our study, RAB proteins RAB11B, RAB7A, RAB5B and RAB7A had differential effect on the release of EVs, specifically RAB7A showing release of high levels of EVs in response to diabetic environment.

In order to dissect out the role of RAB7A in the EV release, we analysed the expression of RAB7A in placental sections obtained from NGT and GDM patients using immunohistochemistry. We identified that RAB7A expression was not significantly different in the sycytiotrophoblast cells between NGT and GDM patients. However, in GDM controlled by diet alone, RAB7A expression was upregulated in endothelial cells and downregulated in Hofbauer cells compared to NGT and GDM controlled by insulin. Hence, RAB7A expression in placenta might be altered in response to the pathology of GDM, but specific role of this protein in trophoblast cells in diabetic pathology is still not clear.

In summary, this study presented evidence of a differential release of a heterogeneous population of EVs from models of placental cells that have been used to represent different spatial and physiological stages of the placenta. Proteins associated with the biogenesis, and trafficking/release of small EVs might be playing key roles in the mediating the response of placental cells to diabetic environment. The physiological context could indicate that changes in the maternal microenvironment involving hyperglycaemia target‐specific cells during the development of the placenta, leading to changes in placentation and the development of pregnancy complications.

## AUTHOR CONTRIBUTIONS

**Carlos Palma**: Conceptualization; data curation; formal analysis; investigation; methodology; writing—original draft. **Andrew Lai**: Investigation; methodology; writing—review and editing. **Katherin Scholz‐Romero**: Investigation; methodology. **Haarika Chittoory**: Investigation; methodology. **Benjamin Van Haeringen**: Methodology; validation. **Flavio Carrion**: Investigation; supervision; writing—review and editing. **Aase Handberg**: Investigation; writing—review and editing. **Martha Lappas**: Data curation; formal analysis; supervision. **Sunil R Lakhani**: Conceptualization; supervision. **Amy E McCart Reed**: Investigation; methodology; supervision; writing—review and editing. **Harold David McIntyre**: Formal analysis; supervision; writing—review and editing. **Soumyalekshmi Nair**: Conceptualization; investigation; methodology; supervision; writing—review and editing. **Carlos Salomon**: Conceptualization; formal analysis; funding acquisition; project administration; supervision; writing—review and editing.

## CONFLICT OF INTEREST STATEMENT

The authors declare no conflicts of interest.

## Supporting information




**Supplementary Figure 1**: Short Tandem Repeat (STR) Profiling Analysis of BeWo and HTR‐ 8/SVneo Cells.
**Supplementary figure 2**: Workflow for overexpression of candidate proteins in cells and evaluation of EV release in response to high (25 mM) and low (5 mM) glucose.
**Supplementary figure 3**: Cell lysates with the overexpressed proteins were analysed using western blot A) Images of membranes immunoblotted with antibody against the FLAG protein and GAPDH antibody B) Molecular weight of the overexpressed protein.
**Supplementary figure 4**. Comparison analysis between small versus large EV.
**Supplementary figure 5**. Comparison analysis of Nanoparticle Tracking Analysis of vesicles isolated from HEK293T cells transfected with RAB7A vector.
**Supplementary Table 1**: List of candidate proteins
**Supplementary Table 2**: Ingenuity Pathway Analysis of proteins from BeWo cells in Vesicle Trafficking database.
**Supplementary Table 3**: Ingenuity Pathway Analysis of proteins from HTR8/SVneo in Vesicle Trafficking database.

## Data Availability

The data that support the findings of this study are available from the corresponding authors, C.S. and S.N., upon reasonable request.
