# Supplementary material for: Differential response of placental cells to high D‐glucose and its impact on extracellular vesicle biogenesis and trafficking via small GTPase Ras‐related protein RAB‐7A
Source: J Extracell Biol. 2024 Jan 15;3(1):e135. doi: 10.1002/jex2.135 (PMC11080917; doi:10.1002/jex2.135)
Supplement: Supplementary file 1 — Supplementary Figure 1: Short Tandem Repeat (STR) Profiling Analysis of BeWo and HTR‐ 8/SVneo Cells. Supplementary figure 2: Workflow for overexpression of candidate proteins in cells and evaluation of EV release in response to high (25 mM) and low (5 mM) glucose. Supplementary figure 3: Cell lysates with the overexpressed proteins were analysed using western blot A) Images of membranes immunoblotted with antibody against the FLAG protein and GAPDH antibody B) Molecular weight of the overexpressed protein. Supplementary figure 4. Comparison analysis between small versus large EV. Supplementary figure 5. Comparison analysis of Nanoparticle Tracking Analysis of vesicles isolated from HEK293T cells transfected with RAB7A vector. Supplementary Table 1: List of candidate proteins Supplementary Table 2: Ingenuity Pathway Analysis of proteins from BeWo cells in Vesicle Trafficking database. Supplementary Table 3: Ingenuity Pathway Analysis of proteins from HTR8/SVneo in Vesicle Trafficking database. [file JEX2-3-e135-s001.docx]

BeWo HTR-8/SVneo


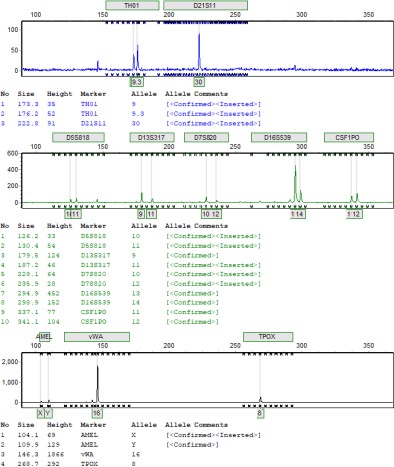

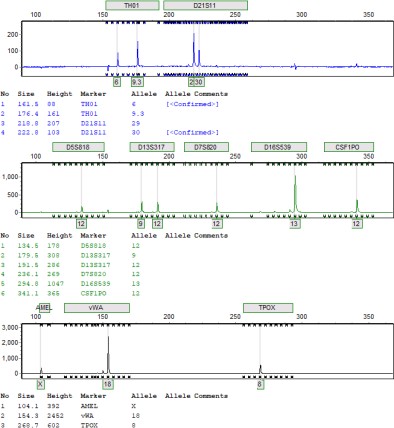


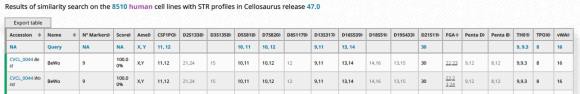

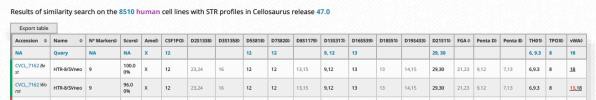


**Supplementary Figure 1: Short Tandem Repeat (STR) Profiling Analysis of BeWo and HTR- 8/SVneo Cells.** This supplementary figure presents the results of Short Tandem Repeat (STR) profiling, a technique used to authenticate and verify the genetic identity of BeWo and HTR-8/SVneo cells. STR profiling is essential for confirming the purity and consistency of these cell lines, ensuring the reliability of the experimental data generated using them. The figure provides valuable insights into the genetic stability and uniqueness of these cell lines, which are crucial factors in maintaining the integrity of research findings.


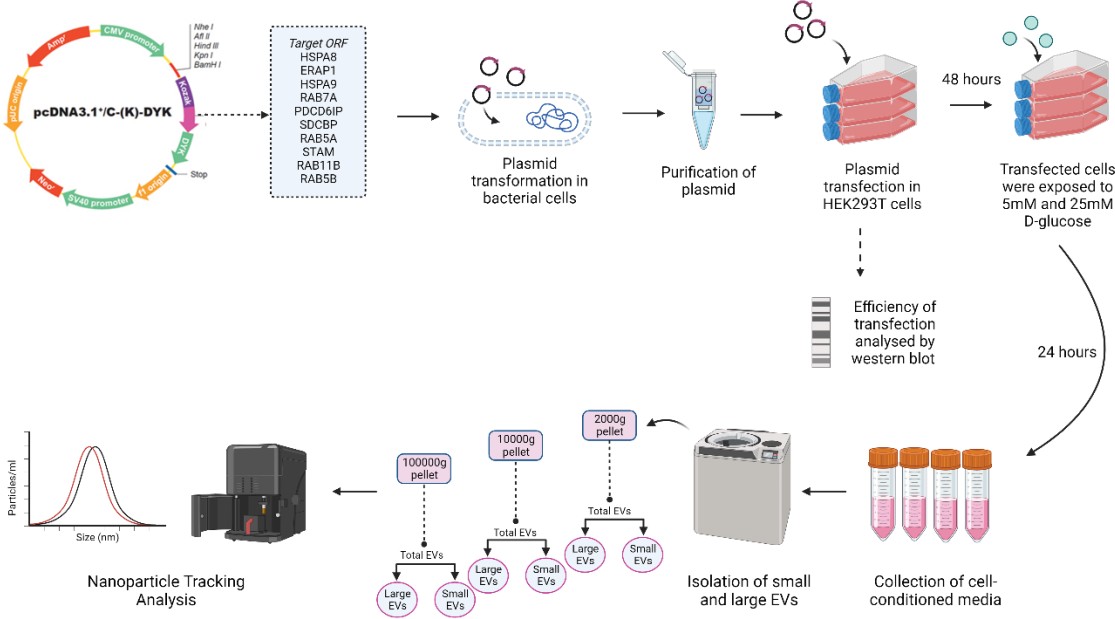


**Supplementary figure 2:** Workflow for overexpression of candidate proteins in cells and evaluation of EV release in response to high (25mM) and low (5mM) glucose. Plasmid containing the target protein ORF and empty vector (as control) were purchased, transformed in bacterial cells, purified from bacterial culture and used for transfection in HEK293T cells. After 48 hours of transfection, the cells were treated with 5mM and 25mM D-glucose for 24 hours. The cell conditioned media was collected and EV isolated by differential centrifugation followed by ultracentrifugation. The 2K, 10K and 100K pellets were analysed by nanoparticle tracking analysis.

# A


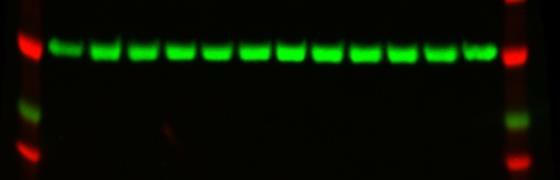

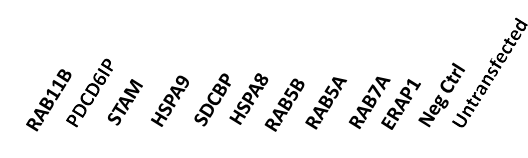

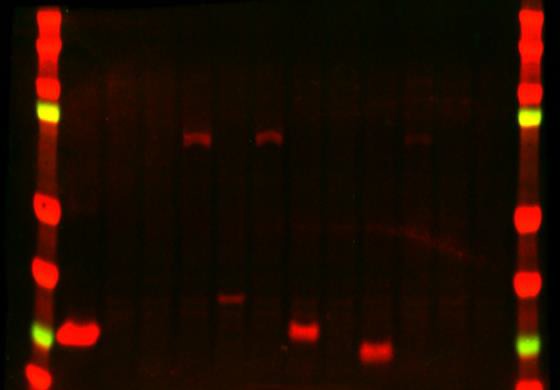


**250**

**150**

**100**

**75**

**50**

**37**

**25**

**20**

**B**

**anti-FLAG**

| **Protein** | **Molecular weight of protein + FLAG tag (DYKDDDDK) in**  **KDa** |
| --- | --- |
| RAB11B | 25.49 |
| PDCD6IP | 97.03 |
| STAM | 60.19 |
| HSPA9 | 74.69 |
| SDCBP | 33.45 |
| HSPA8 | 71.90 |
| RAB5B | 24.71 |
| RAB5A | 24.66 |
| RAB7A | 24.49 |
| ERAP1 | 108.25 |

**Supplementary figure 3:** Cell lysates with the overexpressed proteins were analysed using western blot A) Images of membranes immunoblotted with antibody against the FLAG protein and GAPDH antibody B) Molecular weight of the overexpressed protein.

**anti-GAPDH**


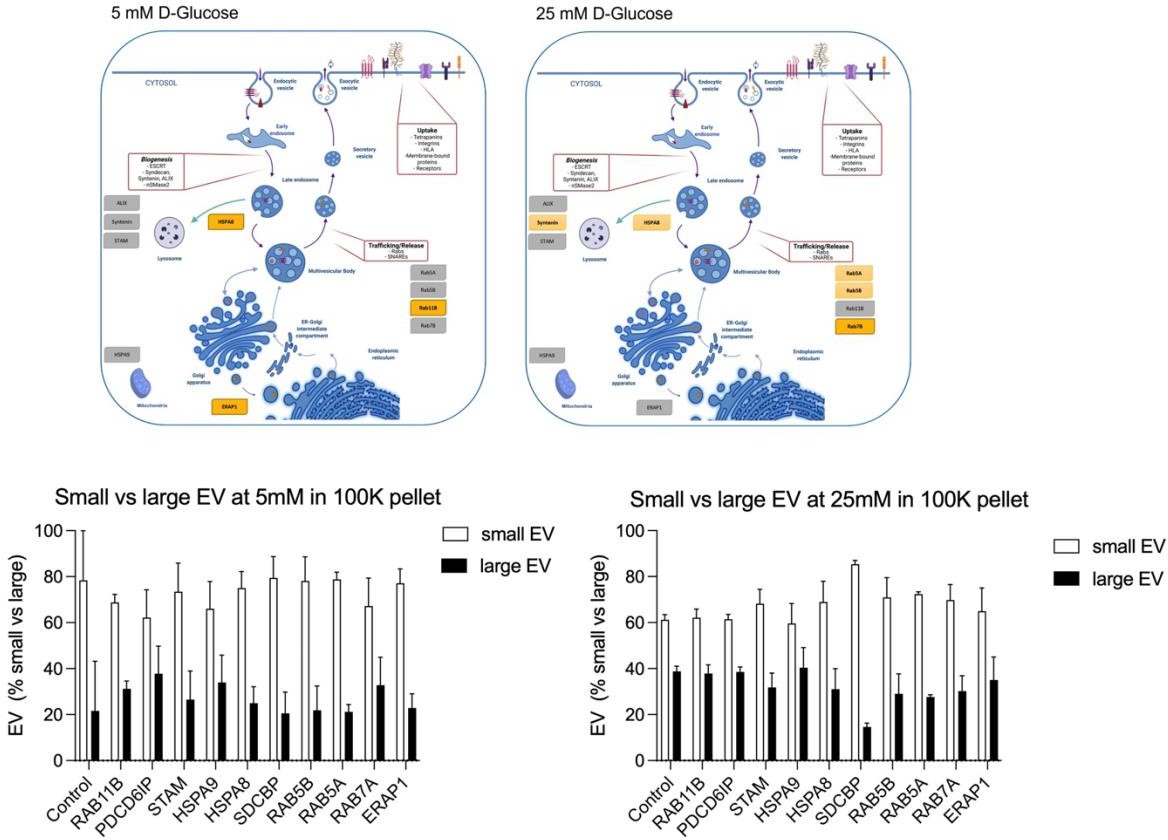


**Supplementary figure 4. Comparison analysis between small versus large EV.** The cell conditioned media was collected and EVs were isolated by differential centrifugation followed by ultracentrifugation from cells transfected with empty vector (control) cultured at 5mM (left) or 25mM (right) D-glucose, and from cells transfected with RAB11B, PDCD6IP, STAM, HSPA9, HSPA8, SDCBP, RAB5B, RAB5A, RAB7A, ERAP1 overexpression vectors. Total EV was quantified by nanoparticle tracking analysis and classified as small EV <200nm, and large EV >200nm. Data is presented as graph bar Mean ± SEM. Top figure show the role of the selected proteins in the biogenesis of EV summarising our results at 5mM and 25mM D- glucose.


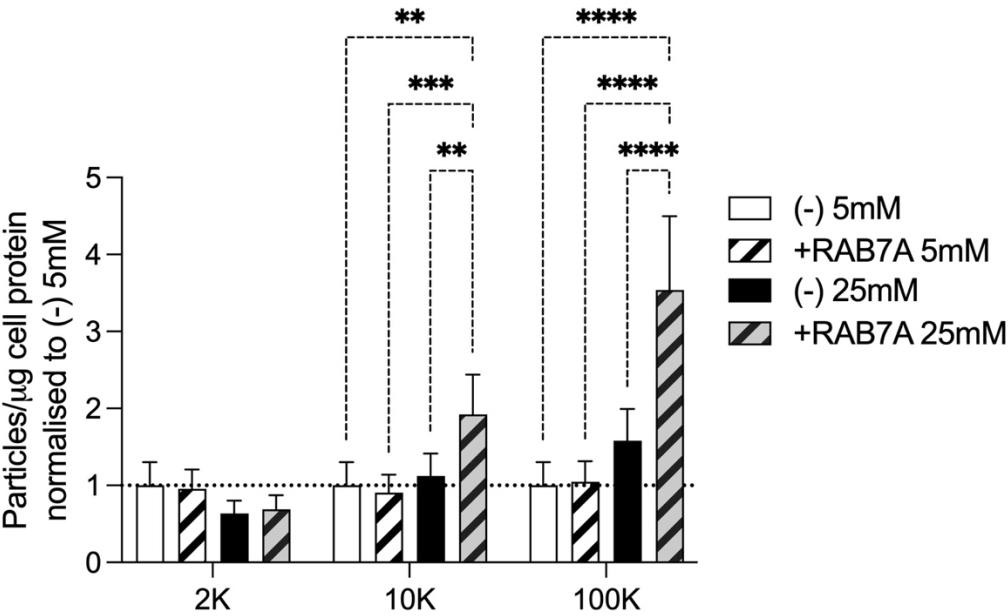


**Supplementary figure 5. Comparison analysis of Nanoparticle Tracking Analysis of vesicles isolated from HEK293T cells transfected with RAB7A vector.** The cell conditioned media was collected and EVs were isolated by differential centrifugation followed by ultracentrifugation from cells without transfection RAB7A overexpression vector (-) cultured at 5mM glucose or 25mM, and with the RAB7A overexpression vector (+RAB7A) at 5mM or 25mM. EV pellet at 2000g (2K), 10,000g (10K), and 100,000g (100K) was analysed by nanoparticle tracking analysis. For each group, i.e., 2K, 10K and 100K, the data is presented normalised to (-) 5mM. at 10K and 100K, **p<0.005, and ***p<0.0005.

**Supplementary Table 1: List of candidate proteins**

| **Symbol** | **Protein** | **Uniprot** |
| --- | --- | --- |
| HSPA8 | Heat shock cognate 71 kDa protein | P11142 |
| ERAP1 | Endoplasmic reticulum aminopeptidase 1 | Q9NZ08 |
| HSPA9 | Stress-70 protein, mitochondrial | P38646 |
| RAB7A | Ras-related protein Rab-7a | P51149 |
| PDCD6IP | Programmed cell death 6-interacting protein (Alix) | Q8WUM4 |
| SDCBP | Syndecan binding protein (Syntenin), isoform CRA_a | G5EA09 |
| RAB5A | Ras-related protein Rab-5A | P20339 |
| STAM | Signal transducing adapter molecule 1 | Q92783 |
| RAB11B | Ras-related protein Rab-11B | Q15907 |
| RAB5B | Ras-related protein Rab-5B | P61020 |

Supplementary Table 2. Ingenuity Pathway Analysis of proteins from BeWo cells in Vesicle Trafficking database.

| **Protein** | **Name** | **UniProt reported function** | **In this study**  **Log Ratio (p-value)** | **Examples related to glucose, apoptosis, cytoskeleton interaction or cytoplasmatic changes in response to stimuli/microenvironment** | **Examples related to placenta** | **Examples related to EVs** |
| --- | --- | --- | --- | --- | --- | --- |
| AIFM1 | Apoptosis-inducing factor mitochondria associated 1 | NADH oxidoreductase and as a regulator of apoptosis. Proapoptotic factor in a caspase-independent pathway in presence of apoptotic stimuli. | -0.384 (0.0122) | Formation of COX-AIFM1_2_ complex, potential roles in oxidative phosphorylation biogenesis and ***apoptosis***. | - | ***Mesenchymal stem cell-derived EVs*** prevent neural stem cell hypoxia by promoting miR-210-3p, which reduce AIFM1 |
| CKB | Creatine kinase B | Involved in energy transduction in tissues where it is required fluctuating large amount of energy. Key regulator of adaptive thermogenesis. | 1.385 (0.00576) | Cr/CK protects the immature brain from hypoxic-ischemic injury by recovering the protein synthesis after ***oxygen-glucose deprivation***. | - | - |
| CYB5R1 | Cytochrome b5 reductase 1 | Desaturation and elongation of fatty acids, cholesterol biosynthesis, drug metabolism | -1.001 (0.0264) | In the early stage of cerebellar granule ***neuronal apoptosis***, there is higher oxidative stress and CYB5R1 has been observed to increase expression.  In a stud of papillary thyroid cancer, increased activity in CYB5R1 has been reported in ***toxic goitre*** patients. | - | - |
| GAPDH | Glyceraldehyde-3-phosphate dehydrogenase | Role in glycolysis and nuclear functions. Evidence suggests GAPDH is involved in microtubule dynamics in the early secretory pathway. Participates in transcription, RNA transport, DNA replication and apoptosis. | -0.25 (0.0434) | ***GAPDH inhibition*** ***increased glucose*** in an *in vitro* model of diabetic retinopathy. Concomitant action of pro-inflammatory cytokines contributes to metabolic dysregulation.  An in vitro study of drug resistance induced by ***high glucose*** showed an association with elevated expression of metabolic enzymes, GAPDH being one of them.  An in vitro maturation of bovine oocytes showed that ***GAPDH is upregulated*** in low oxygen tension when there is a ***low glucose*** availability.  Serum GAPDH levels were significantly lower in ***T2DM patients*** with ***Acute Coronary Syndrome*** than in those without ACS.  GAPDH_Ox_ transfers S-glutathionylation to Sirtuin-1 leading to ***apoptosis.*** | mRNA copy number of ***GAPDH*** in normotensive ***pre-term, severe preeclamptic and term*** placenta does ***not change*** between samples and across gestation.  The ***deamidation*** of ***glyceraldehyde-3-phosphate dehydrogenase*** by Transglutaminase 2 promotes ***trophoblastic cell fusion*** tested in BeWo cell culture by interaction with F-actin and the following rearrangement of cytoskeleton. | Elevated in isolated ***salivary exosomes*** of human papillomavirus -driven oropharyngeal cancer.  ***Biogenesis*** of intraluminal vesicles in endosomal compartments ***requires GAPDH***.  EVs released during Leishmania infection are enriched in (Lm)***GAPDH*** which posttranscriptionally ***inhibits TNA-α*** in macrophages. |
| HSPA8 | Heat shock protein family A (Hsp70) member 8 | Protection of the proteome from stress, pivotal role in the protein quality control system. | 0.181 (0.00862) | HSPA8 decreased in ***Type 2 Diabetes***.  HSP8 is a clathrin-uncoating ATPase during clathrin-mediated ***endocytosis***.  Required in the ubiquitin-***proteasome*** pathway.  HSPA8 inhibits fast axonal transport, whereas supports slow axonal transport by interacting with Kinesin-1.  HSPA8 acts as a ***cellular receptor*** in cases of viral infection, and it has been shown to ***increase after rotavirus infection***.  ***Glucose*** levels activate SLC35B4 expression, separately it has been reported that its knockdown results in the underexpression of HSPA8.  Induced overexpression has been linked to ***attenuated blood pressure*** in PE models, ***promote proliferation and migration, and inhibit apoptosis***. | HSP70 has been detected in high concentration in the amniotic fluid of Intra-amniotic infection, histologic chorioamnionitis, and term parturition women. Presumably as a defence mechanism. | *Hspa8* was one of the top 10 hub genes in EVs from astrocyte and microglia, and although HSPA8 hasn’t been linked directly, it is known that these EVs are involved in neurodegenerative diseases. |
| HSPA9 | Heat shock protein family A (Hsp70) member 9 | May play a role in the control of cell proliferation and cellular aging | -0.185 (0.0204) | Inhibition of IP_3_R–GRP75 (HSPA9) –VDAC1 complex protects against ***programmed necrosis*** (rat / ROS study).  HSPA9 is overexpressed in ***bladder cancer tumor***.  HSPA9 is upregulated in ***breast cancer***, promotes ***proliferation***, ***metastasis and angiogenesis***. It has been also observed to promote the endothelial-to-mesenchymal transition through ***Wnt/β-Catenin***.  In a hepatocarcinoma cell line, it has been shown that overexpression of HSPA9 decreased ***apoptosis*** rate and partial cytoplasmatic distribution of apoptin. Downregulation results in apoptosis. | *HSP70 has been detected in high concentration in the amniotic fluid of Intra-amniotic infection, histologic chorioamnionitis, and term parturition women. Presumably as a defence mechanism. | Mortalin is elevated in exosomes isolated from breast, ovarian, prostate, hepatic, gastric and colon cancer. |
| HYOU1 | Hypoxia up-regulated 1 | Cytoprotective cellular mechanisms triggered by oxygen deprivation. Suppression is associated with accelerated apoptosis. | -0.109 (0.0273) | In diabetic nephropathy, HYOU1 is increased and it is not observed changes in proapoptotic genes. If the hyperglycaemic condition continues, the ER stress induces a protective and adaptative unfolded protein response. The persistence of the condition may lead to ***apoptosis.***  HYOU1 is upregulated in a ***fibrotic kidney fibroblast model***. Normal kidney fibroblast exposed to ***high glucose*** change phenotype to a fibrotic–like with activation of oxidative stress and ER-stress pathways. | ***Heat-stress*** during pregnancy leads to an ***increase*** of corticosterone and the expression of heat shock protein 90 beta family member 1 (hsp90b1), ***hypoxia up-regulated 1*** (hyou1) and corticotropin-releasing hormone receptor 1 enriched in response to glucocorticoids in the fetal brain (murine model). | Exosomes released in acidic microenvironment by ***human melanoma lines*** contain among other proteins HYOU1. The presence of HYOU1 correlates with a poor prognosis due to its role in ***migration and invasion***, therefore, metastasis. |
| IGF2BP1 | Insulin-like growth factor 2 mRNA binding protein 1 | During cellular stress, such as oxidative stress or heat shock, stabilizes target mRNAs that are recruited to stress granules. | 0.339 (0.0185) | IGF2BP1 is highly expressed in Non-small cell lung cancer cells treated with ***high glucose***. Knockdown of IGF2BP1 inhibits ***proliferation, migration and invasion*** and induces cell cycle arrest and ***apoptosis.***  IGF2BP1 is upregulated in hyperthermia resistant colorectal cancer cells and stabilise Lactate Dehydrogenase A mRNA and promote ***glucose metabolism.*** Inhibition of IGF2BP1 sensitized HT resistant cells to the treatment. | Overexpression of IGF2BP1 promotes migration, invasion and proliferation, and suppresses apoptosis. miR-423-5p is highly expressed in PE and targets IGF2BP1 in HTR-8/SVneo cells. | EVs from melanoma overexpressing IGF2BP1 accelerate EV-induced metastasis, opposite to IGF2BP1 knockdown. The knockdown evidenced no changes in size, the number of vesicles or RNA/protein concentration but differential expression of certain mRNAs, proteins and miRNAs. |
| LMAN2 | Lectin, mannose-binding 2 | Intracellular lectin in the early secretory pathway | -0.376 (0.00838) | LMAN2 has been observed to display a gradual decrease across grades of severity in ***Congenital anomalies*** of the kidney and the urinary tract.  LMAN2 interacts with ***Guanylyl cyclase C*** in the same sites allows GC-C to fold and bind ligand. This interaction depends on the glycosylation of these sites. ***Glycosylation*** is an altered process in many diseases.  In addition to ***anterograde traffic***, it is involved in ***glycan-dependent quality control function*** in the Golgi. | - | LMAN2 regulates exosome cargo by affecting the ***trafficking of GPRC5B*** in the Golgi complex for ***exosome release*** without affecting GPRC5B trafficking to the plasma membrane. |
| NAE1 | NEDD8 activating enzyme E1 subunit 1 | Overexpression of NAE1 causes apoptosis through deregulation of NEDD8 conjugation | 0.625 (0.033) | ***Inhibition*** of NAE1 by MLN4924 induces caspase-dependent apoptosis. | Exosomes released from human umbilical cord mesenchymal stem cells improve a model of inflammatory bowel disease (murine model) by increasing, among others, NAE1. | |
| NAPA | NSF attachment protein alpha | Required for vesicular transport between the endoplasmic reticulum and the Golgi apparatus | 0.245 (0.0392) | α-SNAP has a crucial role in the ***exocytosis*** of ***insulin*** in dense-core vesicles.  Decreases when ***Pulmonary Arterial Hypertension*** is established after treatment with ***monocrotaline***.  Positive regulator of extracellular matrix adhesion of human epithelial cells via ***focal adhesion assembly,*** and controls ***tight junctions and adherens junctions*** by controlling Golgi-dependent ***maturation and trafficking of β1 integrin and junctional proteins.***  α-SNAP promotes epithelial cell survival. | NAPA is elevated in assisted reproductive technology placenta and seems to affect membrane fusion. | - |
| RACK1 | Receptor for activated C kinase 1 | Promotes apoptosis by increasing oligomerization of BAX and disrupting the interaction of BAX with the anti-apoptotic factor BCL2L | -0.669 (0.0327) | RACK1 is ***downregulated*** in a murine model of ***diabetes***. Glucose promotes dephosphorylation of IRE1alpha by PP2A. This process is mediated by RACK1.  In astrocytes has been shown that the TNF-αR-RACK1-EED complex binds ***nSMase2*** increasing ***ceramide.*** Accumulation of ceramide generates IL-1β, TNF-α and IL-6. This event can lead to neuronal ***apoptosis.***  Evidence reveals a potential function of RACK1 in ***adiponectin*** signalling transduction through interacting with AdipoR1.  Overexpression leads to enhanced ***cell spreading, increased stress fibers and focal adhesions.*** | RACK1 knockdown in BeWo cells destabilised GCM1 which target gene HTRA4, critical for placental cell migration and invasion. | - |
| RHOA | Ras homolog family member A | Mainly associated with cytoskeleton organization, an active state binds to a variety of effector proteins to regulate cellular responses such as cytoskeletal dynamics, cell migration and cell cycle | 0.128 (0.0429) | Overexpression of RhoA increases the ***transport of glucose*** in response to insulin. The opposite effect has been observed in the knockdown of RhoA. It is thought to be a regulator of ***vesicle trafficking machinery***.  ***High glucose*** induces podocyte ***apoptosis*** and decreases the expression and activity of RhoA.  High glucose induces cardiomyocytes ***apoptosis*** via oxidative stress and activates p38MAPK and JNK through RhoA/ROCK pathway. | RND3 is increased in trophoblasts from the villous tissues of patients with recurrent miscarriage and inhibits trophoblast proliferation and migration in HTR-8/SVneo through regulation of RhoA/ROCk1. Apoptosis is controlled through ERK1/2. | ***siRNA-RhoA*** (among others) ***inhibits the internalization*** of EVs in HeLa cells.  In a unilateral ureteral obstruction model, it was shown that EVs derived from bone marrow mesenchymal stem cells contain milk fat globule-epidermal growth factor-factor 8 and can reduce inflammation, oxidative stress, apoptosis partially through RhoA/ROCK pathway. |
| S100A10 | S100 calcium-binding protein A10 | Induces the dimerization of ANXA2/p36 | -0.488 (0.0387) | In gastric cancer, S100A10 ***activates the mTOR*** pathway through annexin A2 (ANXA2) accelerating tumor ***aerobic glycolysis*** and leading to tumor malignant progression. | ***shRNA-S100A10*** ***deregulated*** 37 genes in ***human endometrial epithelial*** and 256 genes in ***stromal cells***. This deregulation affects ***leukocyte extravasation*** signalling and ***angiogenesis*** pathways fundamental ***during implantation*** | - |
| SQSTM1 | Sequestosome 1 | Bridge between polyubiquitinated cargo and autophagosomes | -0.731 (0.00646) | ***Glucose does not affect*** the expression of autophagy components in neurodegenerative diseases models, however, ***trehalose*** strongly upregulates genes, being Sqstm1/p62 one of them.  In a model with adipocyte-specific deletion of Trx2, it was shown that ***ROS and NF-κβ*** increased the accumulation of p62/SQSTM1 and ***recruits damaged mitochondria***.  In liver cancer studies, p/62/SQSTM1 is related to selective ***autophagic degradation*** of ***hexokinase 2 (HK2)***. Also, ***high levels*** of SQSTM1 and HK2 are observed in patients with ***poor prognoses*** of the disease.  In renal cancer cells, ***knockdown*** of SQSTM1 ***reduces cell growth*** and the expression of  ***glycolytic genes positively regulate HIF-1α through mTORC1.*** | ***Selenium-deficient placentas*** (murine model) present an ***increase in H_2_O_2_*** but also an ***increase*** in autophagic substrate ***p62/SQSTM1***. However, this increase is explained because of an ***impairment of autophagy flux*** and a subsequent accumulation of SQSTM1, and autophagy dysfunction.  ***Autophagic machinery*** is ***upregulated*** during the ***differentiation*** of Trophoblast Stem cells | The ***inhibition*** of the autophagy-lysosome pathway (ALP) ***upregulates*** ***α-Synuclein*** association with ***EVs*** in neuronal cells and transfer it to other cells. Also, ***components*** of the ***ALP*** are found as ***EV-cargo***. |
| LGALS3 | Galectin 3 | Involved in acute inflammatory responses | -0.361 (0.0229) | Gal-3 is ***upregulated*** in a model of ***insulin-resistant macrophages***.  In rat brains, Gal3 ***increases vessel density and neuronal survival*** after ischemia/reperfusion injury and ***downregulates*** ***pro-apoptotic*** and ***inflammatory*** genes.  Evidence associates ***circulating galectin-3*** levels and ***GDM*** and might be mediated via ***insulin resistance***. | Gal-3 is ***upregulated*** in ***blood*** and ***placental tissue*** of ***GDM*** women but decreased in cord blood, giving evidence of the protective role of the placenta.  ***High glucose*** concentration and ***hyperglycemia upregulate gal-3 in*** *vitro* (HUVECs) and *in vivo* (diabetic rat model).  ***BeWo cells*** produce and release ***Gal-3*** in response to ***17β-estradiol (E2), progesterone (P4), and human chorionic gonadotropin (hCG),*** inhibiting cell proliferation and i***nducing apoptosis*** of endometrial cells through Integrin-1β.  ***Deficiency*** of Gal-3 in the placenta (murine model) leads to ***placental inflammation and malperfusion,*** and uterine ***NK cells infiltration***. | EVs from human umbilical cord blood ***enriched in Gal-3*** (among other proteins) have an ***immunosuppressive effect in T cells.*** |
| CDK2 | Cyclin dependent kinase 2 | Regulates balance between cellular proliferation, cell death, and DNA repair in human embryonic stem cells. Cyclin E/CDK2 prevents oxidative stress-mediated Ras-induced senescence | 0.713 (0.0388) | Knockout of ***Translationally controlled tumor-associated protein*** (implicated in cell growth and proliferation of pancreatic β-cells as glucose-regulated supportive mechanism) ***decreases the expression of CDK2***. The effect with the regulation of other proteins ends up in a decrease of β-cells proliferation and growth, an ***essential process in adaptation to insulin resistance***.  Murine mesangial cells exposed to ***high glucose*** express p27Kip1, which binds and ***inhibits CDK2*** and leads to ***hypertrophy***, an early hallmark of diabetic nephropathy.  Cardiomyocytes exposed to doxorubicin induce ***phosphorylation by CDK2*** of the ***transcription factor forkhead box O1*** and activating transcription of the ***proapoptotic target*** gene, Bcl-2-interacting mediator of cell death. | Pollutant ***1-nitropyrene induces genotoxic stress*** (partially ROS-mediated) and leads to ***inhibition of placental trophoblasts*** ***proliferation*** and fetal IUGR by decreasing CDK2 (among others) and other downstream proteins.  ***HUVECs*** obtained from ***preeclamptic women*** shows ***downregulation of CDK2–cyclin E*** complexes and an ***increase of the Bax/Bcl-2 ratio*** which ends up in the initiation of the ***apoptotic machinery***. | ***Exosomes*** isolated from Mouse ***mesenchymal stem cells*** have a ***suppressive*** effect on T-cell ***proliferation*** by inducing cell cycle arrest through ***downregulation*** of ***CDK2*** (and downregulation of p27Kip1). |
| CLIC3 | Chloride intracellular channel 3 | Insert into membranes and form chloride ion channels, probably related to control of cell growth | 0.452 (0.0342) | Analysis of microarray of ***gastric cancer specimens*** showed a negative correlation between expression of CLIC-3 and pathological tumor depth (related to prognosis). *In vitro* experiment in a human gastric cancer cell line (MKN7) indicated that ***proliferation was accelerated*** in a ***knockdown of CLIC-3.***  ***CLIC-3*** is abundant in cancer-associated fibroblasts and exhibits a ***glutathione-dependent oxidoreductase activity*** which reduces transglutaminase-2, finally driving ***angiogenesis and cancer progression***. | CLIC3 is expressed in human placentae and fetal membranes but ***mRNA and protein*** are ***increased*** ***in FGR, PE*** and concomitant cases. | - |
| RAN | RAN, member RAS oncogene family | RAN controls cargo loading and release by transport receptors in the proper compartment and ensures the directionality of the transport | -0.306 (0.0472) | ***Overexpressed*** in Oral squamous cell ***carcinoma***. ***Inhibition*** of RAN leads to a ***decrease in cell proliferation and also apoptosis and cell cycle arrest***.  ***Ran*** transgenic mice showed T cells with compromised ***activation marker expression***, ***lymphokine secretion***, and ***proliferation*** upon T cell receptor activation (*in vitro*). *In vivo*, it was observed a defective delayed-type hypersensitivity. | - | - |
| SF3B3 | Splicing factor 3b subunit 3 | Component of the splicing factor SF3B complex, part of the spliceosome | 0.174 (0.00215) | Factors are shown to be ***highly mutated*** in ***hematological malignancies***, however, ***SF3B3 shows only one single nucleotide polymorphism*** (aminoacid substitution).  In a breast cancer study, ***SB3B3*** is highly ***upregulated*** in ***Oncotype DX high-risk***. Results compared against breast cancer gene expression data sets show a positive correlation with overall survival (ER-positive). |  | In an anti-cancer drug study, treatment with human cerebral endothelial cell-derived exosomes carrying elevated ***miR-214*** combined with oxaliplatin or sorafenib showed to ***reduce*** protein levels of ***SF3B3*** in hepatocellular carcinoma. It is known that SF3B3 is involved in drug resistance and cancer cell proliferation. |
| ITGB1 | Integrin subunit beta 1 | Integrins are heterodimers used by animal cells to link the extracellular matrix and the actin cytoskeleton. | 0.186 (0.0338) | ***Epidermal growth factor protects*** podocytes from ***high glucose*** treatment. Informatic analysis revealed that the ***ITGB1 hub gene*** seems to be associated with the protection mechanism.  To alleviate the progression of ***Diabetic retinopathy***, ***lysine acetyltransferase 1*** induce expression of YTHDF2 and triggers ***instability of ITGB1 mRNA***. | ***In a PE study, miR-29b*** regulates the ITGB1 gene (among others) which ***induces apoptosis and inhibits invasion and angiogenesis o***f trophoblast cells.  ***Overexpression*** in ***HTR-8/SVneo*** cells enhanced cell ***proliferation, migration, and invasion, reduced cell apoptosis*** through ***activation of the PI3K/Akt pathway.*** | In a model of ***early cerebral ischemia*** (oxygen-glucose deprivation by 3 hours), changes were observed in the ***miRNA content of exosomes*** derived from blood-brain barrier endothelial cells. ITGB1 was one of the 24 membrane proteins targeted by these miRNA. |
| LAMP1 | Lysosomal associated membrane protein 1 | Role in lysosome biogenesis, autophagy, and cholesterol homeostasis | -0.456 (0.00776) | Increasing the concentration of ***glucose*** leads to a ***decrease of mRNA and protein*** expression of mTOR and also ***LAMP1*** in renal epithelial cells.  In a ***silicosis study***, **trehalose** induces an ***increase of LAMP1*** and a decrease of LAC3 leading to an ***increase*** in activity of the ***autophagy-lysosomal system in alveolar macrophages.*** | In ***hypoxia-exposed trophoblasts***, there is an **inhibition** of ***transcription factor EB*** which impacts ***LAMP1***, LAMP2, and CTSD (cathepsin D). The same ***impaired lysosomal biogenesis and autophagy*** is observed in PE placenta. | Transplanted ***mesenchymal stem cells release apoptotic bodies*** which increase expression of ***transcription factor EB***, regulating the downstream expression of LAMP1. This process ***promotes angiogenesis*** via regulating ***autophagy*** in endothelial cells. |
| SLC2A1 | Solute carrier family 2 member 1 | Facilitative constitutive glucose transporter | -0.372 (0.0105) | ***Imatinib downregulates*** expression of ***GLUT-1*** and other glycolysis-related molecules in gastrointestinal stromal tumors. ***Imatinib-resistant*** cells show an ***increase*** in these ***glycolytic molecules***.  Blastocysts from early cleaving embryos there is a ***high expression*** of genes involved in ***aspartame and carbohydrate metabolism***, including ***Slc2a.*** | ***GLUT1 upregulation*** is observed during ***decidualization*** and ***increases*** the levels of ***cAMP*** which induces decidualization (establishment of pregnancy). However, this increase is suppressed by knockout of CCAAT enhancer-binding protein β (C/EBPβ) and Wilms' tumor 1 (WT1) | Macrophages with ***ERCC1-XPF DNA repair defect subject to continuous DNA damage release Er1^−/−^- EVs, these EVs*** promote ***GLUT1 accumulation, insulin-independent glucose uptake***, recruitment of macrophages in parenchymal tissues and ***enhancement glucose tolerance*** in wild-type animals. The glucose uptake leads to inflammation through activation of the mTOR signalling pathway. |
| TLN1 | Talin 1 | Probably involved in connections of major cytoskeletal structures to the plasma membrane | 0.577 (0.0449) | ***TLN1*** is ***elevated*** in the serum of ***hepatocellular carcinoma patients***.  TLN1 is known to be a mechanosensitive component of adhesion complexes. Interaction with CDK1 is thought to be a mechanism in which the microenvironment can control cell division in multicellular organisms. | - | ***EVs*** obtained from patients with ***Myalgic encephalomyelitis / chronic fatigue syndrome*** show ***abundant talin-1***, filamin-A, and 14-3-3 family proteins, which are involved in ***focal adhesion, actin skeletal regulation, PI3K-Akt signalling pathway, and Epstein-Barr virus infection***. |

Supplementary Table 3. Ingenuity Pathway Analysis of proteins from HTR8/SVneo in Vesicle Trafficking database.

| **Protein** | **Name** | **UniProt reported function** | **In this study**  **Log Ratio (p-value)** | **Examples related to glucose, apoptosis, cytoskeleton interaction or cytoplasmatic changes in response to stimuli/microenvironment** | **Examples related to placenta** | **Examples related to EVs** |
| --- | --- | --- | --- | --- | --- | --- |
| ARRB1 | Arrestin beta 1 | Acts as an endocytic adapter and is involved in the internalisation of multiple receptors (desensitisation). Scaffold for MAPK pathways | -0.327 (0.0336) | The adaptive β-cell replication (mass expansion) under an obesogenic environment to maintain glucose homeostasis needs β-Arrestin-1.  GRK2/ β-Arrestin-1 overexpression attenuates glucose-dependent insulinotropic polypeptide-induced insulin release and cAMP production. | First-trimester placental villi exhibit a supportive mechanism for programmed development that includes regulation of apoptosis and cell cycle progression, being ARRB1 one of the components.  Mechanosensitive AT1-B2 receptor heteromers can be downregulated by ARRB1 improving PE symptoms. | In a study of potential implications of cell-based therapy, it was found that the cargo of EVs isolated from porcine adipose tissue-derived mesenchymal stem cells expresses, among other molecules, Golgi apparatus genes ARRB1 and GOLGA4. |
| CTBP2 | C-terminal binding protein 2 | Involved in the corepression of several transcription regulators and brown adipose tissue differentiation. | -1.046 (0.0379) | CtBP2 play a sensing role and controlled FoxO1 in order to suppress hepatic gluconeogenesis, however, this activity is defective in the obese liver. diabetes and hepatic steatosis in obesity can be improved by activation of CtBP2.  Combination of NADH increase, a reduced expression of CtBP, or transfection with a NAD(H) insensitive CtBP leads to decreased NF-κB transcriptional activity and pro-inflammatory gene expression in macrophages and microglia. | Ctbp2-null mice suffer aberrant extraembryonic development which leads to dead whereas Ctbp1 has no same effect. |  |
| ERAP1 | Endoplasmic reticulum aminopeptidase 1 | Responsible for peptide trimming, and potentially involved in blood pressure regulation. | -0.535 (0.02) | In a study of ankylosing spondylitis, the different alleles of ERAP1 and its interaction (or not) with HLA-B27 do not modify the level of ER stress. | Preeclamptic placental tissues have a high expression of ERAP1. The expression is also increased by hypoxia in HTR8/SVneo. | ERAP1 has a domain that allows the binding to exosome and it has been proved that activate exosome-dependent phagocytosis and NO synthesis of the cells |
| GAK | Cyclin G associated kinase | Seems to be involved in the uncoating of clathrin-coated vesicles by Hsc70 in non-neuronal cells (auxilin-like) | -1.781 (0.0426) | Evidence indicates that in the proliferation of cancer cells, GAK phosphorylates Clathrin heavy chain (CHC)-T606, which later translocates to the nucleus. CHC and GAK also interact with PLK1, the activation of this axis may participate in proliferation. | - | - |
| GRN | Granulin precursor | Growth factor involved in inflammation, wound healing and cell proliferation but also regulators of lysosomal trafficking of proteins and the activity of lysosomal enzymes. | 1.187 (0.0354) | Loss of GRN function in microglia leads to neurodegeneration and reduce cerebral glucose metabolism. This is through activation of the molecular signature for microglial neurodegenerative phenotype but also suppression of gene characteristics for homeostatic microglia.  Progranulin is related to obesity and insulin resistance and physical components of metabolic syndrome. | In PE and FGR cases, it was found that villous trophoblasts have higher protein expression of progranulin but mRNA. | Grn-/- mice model lysosomal abnormalities observed in frontotemporal dementia. Brain EVs in this murine model and patients were elevated, and the EV protein profile was altered. |
| HSPA5 | Heat shock protein family A (Hsp70) member 5 | Endoplasmic reticulum chaperone involved in the degradation of misfolded proteins and auxiliary role in post-translational transport of small presecretory proteins across the endoplasmic reticulum | -0.171 (0.0171) | Astrocytes under recurrent acute low glucose exposure increase the expression of endoplasmic-reticulum stress gene-related (unfolded protein response) such as HSPA5. In cases of recurrent low glucose, this response decreases, suggesting attenuated ER stress.  In biopsies samples of established diabetic nephropathy HSPA5 (and HYOU1) increase. In addition, no changes were observed in proapoptotic genes. If the hyperglycaemic condition continues, the ER stress induces a protective and adaptative unfolded protein response although the persistence of the insult may result in apoptosis. | Prolactin family 8, subfamily a, member 2 targeted several genes. In null Decidual–placental-embryonic tissue, Hspa5 is upregulated evidencing an increase in endoplasmic reticulum stress response. | HSPA5 is considered among proteins of the surface composition of large EVs |
| MAP1B | Microtubule-associated protein 1B | Positive cofactor in DAPK1-mediated autophagic vesicle formation and membrane blebbing | 1.112 (0.0181) | Using mouse embryonic fibroblasts, it was shown that MAP1B is cleaved by Calpain-10, a susceptibility gene for type 2 diabetes mellitus. In absence of Calpain-10 (knockout), high and low glucose increase insulin secretion. It is thought the process involves problems in the reorganization of actin. | - | Human neuroblasts decreased miRNA expression in neurites. Analysis of the media found extracellular vesicles containing miRNA and enriched in MAP1B. This evidence suggests miRNA as neural plasticity regulators. |
| MAPK3 | Mitogen-activated protein kinase 3 | Involved in cell growth, adhesion, survival and differentiation by regulating transcription, translation, cytoskeletal rearrangements. Also plays a role in the regulation of the endosomal dynamics. | -2.083 (0.046) | The proliferation of interstitial cells of Cajal lineage and gastric organotypic cultures was evidenced under hyperglycemic conditions. This proliferation is by activation of MAPK1 and MAPK3 and downstream ETV1 and KIT.  Using machine learning methods, it was shown that high glucose is the main metabolite impacting the early stage of bladder cancer through cancerogenic genes AKT, EGFR, MAPK3. | Human placenta-derived mesenchymal stem cells are normally exposed to hypoxic conditions. Culture under 5% O_2_ showed HIF-2α bound to the MAPK3 promoter regulating proliferation.  Placental angiogenesis has been proved to rely on MAPK3/1, AKT1, and NOS3/NO activation which is triggered by Endogenous hydrogen sulphide donors derived from trophoblasts. | Prostate cancer cells and EVs isolated from them contain circ_SLC19A1 which target miRNA-497 therefore the subsequent expression of Septin-2 and downstream activation of MAPK3. This control regulates cell growth and invasion.  MAPK3 is activated in cardiomyocytes by treatment with exercise-induced EVs and offering anti-apoptotic protection against oxidative stress. |
| NANS | N-acetylneuraminate synthase | Synthetises N-Acetylneuraminic acid and deaminated neuraminic acid (preventing infections) | -0.084 (0.00576) | In the field of metabolic engineering, it has been developed an *in vivo* active aptazyme-based biosensor responsive to N-acetylneuraminate in a dose-dependent fashion. | - | - |
| PDCD6IP | Programmed cell death 6 interacting protein | Involved in endocytosis, multivesicular body biogenesis, membrane repair, cytokinesis, apoptosis and maintenance of tight junction integrity but also biological processes apoptosis and celroliferation | 0.259 (0.0386) | The intracellular trafficking of amyloid precursor protein relies on Alix and Syntenin-1. The neurotoxic C-terminal fragment (CTFβ) has a similar secretion pathway to small EVs. | Extracellular vesicles isolated from syncytiotrophoblast are positive for PLAP but also for EV markers CD63 and ALIX. | ESCRT machinery requires ATP to disassemble ALIX and CHMP4b. when cells are ATP depleted, the machinery gets locked. The disassembly process continues once the ATP is restored.  ALIX and RAB35 activation is produced by treatment with insulin-like growth factor-1 (mimic exercise) and leads to an increase of EV release. |
| RAB7A | RAB7A, member RAS oncogene family | Small GTPase involved in the regulation of endo-lysosomal trafficking | -0.188 (0.0335) | Inhibition of RAB7A improves IGF-1 and HGF signalling in beta cells and increases expression of the growth factor receptors IGF-1R and c-Met. In metabolic stress, it has survival and protective role against apoptosis and autophagy pathways.  In breast cancer studies, proliferation, migration and growth of xenograft tumor are affected by downregulation of RAB7A. | - | In cases of severe traumatic brain injury, EVs isolated from cerebrospinal liquid have an increased expression of Rab7A although time-dependent (delayed). Rab7A negative correlates with EV concentration but not with size. |
| RUVBL2 | RuvB like AAA ATPase 2 | Negatively regulates the expression of ER stress response genes. Potential role in oncogene and proto-oncogene mediated growth induction, tumor suppressor mediated growth arrest and replicative senescence, apoptosis, and DNA repair. | 0.13 (0.0441) | A murine model of liver-specific Reptin knockout shows a decreased body and fat mass, hypoglycaemia and hypolipidaemia explained by a diminished abundance of hepatic mTOR. Deletion of RUVBL2 rescues glucose intolerance, hyperglycaemia, hyperlipidaemia, and hepatic steatosis.  In adipocytes, RUVBL2 is highly expressed and is mainly distributed in the cytosol and it is involved in GLUT4 translocation and glucose uptake stimulated by insulin.  RPAP3 controls UV-induced DNA damage through H2AX phosphorylation. | RuvBl2 is one of the 3 genes expressed in response to high glucose in germ cells. | - |
| SART1 | Spliceosome associated factor 1, recruiter of U4/U6.U5 tri-snRNP | Component of the spliceosome | -0.396 (0.0384) | Hypoxia increases SART1 in epithelial to mesenchymal transition and activation of the NF-κB pathway with a switch of HIF-1α to HIF-2α. The adaptation leads to the enhancement of migration/invasion capacity.  HLA-A24-restricted and tumor-specific cytotoxic T lymphocytes can be induced by a SART1-peptide, and it has been used as immunotherapy in the treatment of esophageal cancer | Tyrosine kinase inhibitor reduces mRNA and protein levels of HIF-1α via SART1. At the same time, SART1 enhances HIF-2α transactivation activity leading to the production of proangiogenic factors and cytokines and promoting HUVEC proliferation (tested in Human renal adenocarcinoma cell line and renal proximal tubular epithelial cell line). | - |
| UCHL1 | Ubiquitin C-terminal hydrolase L1 | Ubiquitin-protein hydrolase (ubiquitin precursors and ubiquitinated proteins) | 0.28 (0.0233) | - | - |  |
